# Compost application more strongly shapes soil fungal than bacterial communities across arable fields

**DOI:** 10.1093/ismeco/ycag259

**Published:** 2026-09-10

**Authors:** Florian Walder, Kyle Hartman, Anna Edlinger, Chantal Herzog, Alain Y Valzano-Held, Sana Romdhane, Laurent Philippot, Samiran Banerjee, Marcel G A van der Heijden

**Affiliations:** Agroecology and Environment, Agroscope, Zurich 8046, Switzerland; Agroecology and Environment, Agroscope, Zurich 8046, Switzerland; Agroecology and Environment, Agroscope, Zurich 8046, Switzerland; Wageningen Environmental Research, Wageningen University & Research, Wageningen 6708 PB, The Netherlands; Agroecology and Environment, Agroscope, Zurich 8046, Switzerland; Agroecology and Environment, Agroscope, Zurich 8046, Switzerland; Department of Evolutionary Biology and Environmental Studies, University of Zurich, Zurich 8057, Switzerland; INRAE, UMR Agroécologie, Université Bourgogne Europe, Institut Agro Dijon, Dijon 21000, France; INRAE, UMR Agroécologie, Université Bourgogne Europe, Institut Agro Dijon, Dijon 21000, France; Department of Microbiological Sciences, North Dakota State University, Fargo, ND 58102, United States; Agroecology and Environment, Agroscope, Zurich 8046, Switzerland; Department of Evolutionary Biology and Environmental Studies, University of Zurich, Zurich 8057, Switzerland

**Keywords:** soil microbiome, organic amendments, compost application, fungal diversity, co-occurrence networks, soil organic carbon, aggregate stability

## Abstract

Organic amendments such as compost are widely promoted for enriching soil microbial communities and supporting key soil functions, yet their effects across diverse farming systems and pedoclimatic conditions remain poorly understood. Most evidence originates from controlled experiments that cannot account for the variability inherent in real agronomic settings. Here, using data from 56 commercially managed wheat fields under different fertilizer regimes (compost, solid digestate, or manure) across Switzerland, we show that compost application reshapes soil microbial communities under real farming conditions. Compost increased fungal diversity, shifted fungal community composition, and markedly enhanced fungal network complexity (+50%), exceeding effects of the overall management system (organic versus conventional). While compost effects were strongest for fungal communities, 13 bacterial and nine fungal taxa also increased in abundance following frequent compost application. We further identified a distinct microbial cluster enriched with compost-responsive taxa, including thermophilic taxa from both bacterial (Thermoactinomycetaceae) and fungal (*Thermomyces*) lineages characteristic of composting processes, suggesting establishment of compost-derived microbes in agricultural soils. In contrast, solid digestate from biogas plants had weaker and more variable effects on soil microbial communities. Structural equation modelling suggested that compost was associated with higher soil respiration mediated primarily through soil physicochemical properties and to a lesser extent microbial community traits. Together, the results provide evidence that compost application structures soil microbial communities across a wide range of pedoclimatic conditions and management backgrounds within Swiss agroecosystems, supporting its role as an effective practice for strengthening microbially mediated soil processes and promoting sustainable soil management.

## Introduction

Organic amendments are fundamental to sustainable soil management, aiding in the replenishment of soil organic carbon (SOC)—a vital element of soil health, which is often depleted by intensive agriculture. SOC enhances soil structure, increases water retention, supports nutrient cycling, regulates greenhouse gases, and sustains active and diverse biological communities [1]. Historically, manure and slurry were commonly used as fertilizers in smallholder mixed farming systems, enabling the regular application of organic amendments. However, with the introduction of mineral fertilizers, the depletion of SOC has intensified, and the addition of organic amendments is regarded as essential for restoring SOC levels and supporting long-term soil health [2]. In this context, alternatives to farmyard manure, such as compost and solid digestate (the solid fraction of digestate produced by anaerobic digestion of biogenic waste in biogas plants), have gained increasing interest. Compost has become particularly popular due to its recognized benefits for soil health. Indeed, various experimental studies have shown that compost application has great potential for enhancing SOC, improving soil water-holding capacity, and increasing nitrogen use efficiency, while supporting soil microbial communities [3–7].

Soil microbes drive decomposition, nutrient cycling, pathogen suppression, and aggregate formation, underpinning ecosystem functioning and productivity [8–11]. Promoting these communities through agricultural management offers potential for enhancing soil health [12]. Compost application has been shown to support diverse and beneficial microbiomes [13, 14]. Microbially rich composts in particular can induce enduring shifts in soil microbial diversity, community composition, and structure [15, 16] and enhance microbial activity, leading to improved nutrient cycling and accelerated decomposition of organic matter [4, 6].

While it is unclear whether microbiome shifts actively drive compost’s beneficial effects on soil health, or whether they primarily result from compost-induced changes in soil physicochemical properties [17], a more fundamental knowledge gap concerns the generalizability of compost effects across diverse contexts. Most existing studies have focused on single-site field experiments directly comparing compost-amended and unamended treatments [4, 6, 15], leaving compost effects across diverse management and pedoclimatic conditions poorly understood. Yet the outcome of compost application is unlikely to be uniform across such contexts, as it depends on both the characteristics of the compost itself—its type, quality, and quantity [13, 15, 18]—and on the process of community coalescence, in which the compost-borne microbiome interacts with a resident community shaped by the pedoclimatic conditions, cropping history, and broader management context of each field, including tillage, herbicide use, and crop cover [4, 6, 15, 19, 20].

To address this gap, we surveyed a network of 56 commercial farms spanning diverse pedoclimatic and management conditions, including both conventional and organic systems with a range of fertilization, agrochemical, tillage, and crop rotation practices. Across this network, we investigated whether compost application shapes soil microbiota, compared with a manure-based reference fertilization regime. Given the rise of biogas plants, we additionally included farms applying solid digestate. We specifically analysed effects on bacterial and fungal community composition, diversity, and network complexity. This study builds upon earlier work on the same network, where we found that compost enhanced basal respiration and cation exchange capacity, with a trend towards higher SOC stocks [7]. We hypothesize that (i) compost and digestate application shape soil microbial community structure, diversity, and network complexity under real farming conditions across a heterogeneous farm network; and (ii) the magnitude and nature of these effects differ between compost and digestate application.

## Materials and methods

### Farm network

The study was conducted on 56 Swiss farms, as detailed in Edlinger *et al.* [7]. These farms span a wide pedoclimatic gradient within Swiss temperate agroecosystems, encompassing substantial variation in soil texture, pH, bulk density, mean annual temperature, and precipitation. The selected farms were based on their management systems (conventional or organic farming) and fertilization regimes, which included frequent applications of compost or solid digestate (at least one application in the last five years), or a reference treatment receiving neither. The groups comprised: conventional fields with compost (*n* = 17), conventional fields with solid digestate (*n* = 9), conventional reference fields without these applications (*n* = 13), organic fields with compost (*n* = 9), and organic reference fields without compost application (*n* = 8). Reference fields received standard Swiss mixed fertilization including substantial carbon inputs via manure and slurry, such that fertilizer regimes were comparable in total carbon input but differed in the qualitative characteristics of the organic amendment applied. We additionally recorded management data, including carbon inputs from each organic fertilizer, crop-rotation richness, soil cover and leys, along with mineral nitrogen (N) and phosphorus (P) inputs (refer to Edlinger *et al.* [7] for more details). The date of the last compost fertilizer application was recorded during farmer interviews; where an exact date was unavailable, it was estimated from the reported timing relative to field operations or, where only a month or year was reported, taken as the midpoint of that period—sufficient to place each application within the correct growing season relative to sampling. Furthermore, we calculated indices for tillage, herbicide, and fungicide use based on the number of events, depth, or agrochemical products, which were scaled and averaged.

#### Soil sampling and analysis

Soil was sampled once per field between May and June 2017 by collecting ten 0–20 cm cores within a 10 m radius and combining them into a single composite sample. Samples were sieved (2 mm), then air-dried for physicochemical analyses or stored at −20°C for DNA extraction. Dried subsamples were analysed for texture, CEC, total N, organic C, available K, total P, and pH following Swiss Reference Methods [21]; bulk density was determined on four undisturbed 100 cm^3^ cores at 10 cm depth. Aggregate stability was assessed by wet sieving of undisturbed cores to calculate the mean weight diameter [22]. The same soil samples were also used to assess soil basal respiration according to the Swiss Reference Methods [21].

#### Microbial diversity and community structure

Prokaryotic (16S rRNA) and fungal (ITS) communities were profiled following sequencing protocols described by Romdhane *et al.* [23] and Banerjee *et al.* [24]. DNA was extracted from composite soil samples, quantified, and amplified; bacterial libraries were sequenced using Illumina, and fungal libraries were sequenced using PacBio. Reads underwent quality filtering, demultiplexing, OTU clustering at 97% similarity, and taxonomic assignment, with low-abundance OTUs removed. Putative functions were inferred from taxonomy via the microeco R package [25]: bacterial OTUs were annotated with FAPROTAX [26] and fungal OTUs with FUNGuild [27] (see Supplementary Material and Methods).

α-Diversity, Shannon index and evenness [28], were calculated after rarefying to the smallest sequencing depth (15 759 reads for bacteria; 1597 for fungi; Fig. S1) and tested by ANOVA. Community structure was visualized using principal coordinates analysis (PCoA) and constrained analysis of principal coordinates (CAP), and tested by PERMANOVA on Bray–Curtis distances of log₂(x + 1)-transformed OTU counts [29]. In addition, we assessed dispersion effects [29] and performed pairwise group comparisons between fertilizer regimes and management systems [30]. Multivariate ANOVA and PERMANOVA models were specified as in Schmid *et al.* [31], with collinearity among the variables tested beforehand. The general model formula was response ~ spatial eigenvectors + climatic variables + soil physicochemical properties + fertilizer regime + management system + management indices. As each field was sampled once, the model included no random effect for site. Spatial eigenvectors, climate, and soil variables were entered first, followed by fertilizer regime (compost, digestate, or reference), management system (organic or conventional), and management indices (e.g. organic C input). The spatial eigenvectors were derived from principal coordinates of neighbour matrices [32]. Residuals were checked for normality and homoscedasticity; log or Box–Cox transformations were applied as necessary. Effect sizes of individual predictors are reported as the percentage of total sum of squares (%SS), providing a direct comparable measure of each predictor’s unique contribution within the sequential model, with effect direction indicated by standardized coefficients. Partial *R*^2^ values were calculated using a custom function and are reported to decompose variance among environmental variables, management system, and fertilizer regime, with each contribution calculated after partialling out all other predictors in the model.

#### Identification of differentially abundant OTUs and enrichment of taxonomic groups

The study analysed variation in the relative abundances of individual OTUs and the enrichment of bacterial or fungal taxonomic groups using non-rarefied data [33], with DESeq2 normalization [34]. Differential abundance was tested in DESeq2 using pairwise contrasts—compost vs. reference and digestate vs. reference—with the same spatial, climatic, and soil physicochemical covariates as the community-level analyses included in the model. OTUs were classified as compost-, digestate-, or reference-associated based on the direction and significance of the respective contrast. *P*-values from all OTUs were adjusted for multiple testing (false discovery rate, FDR [35]). OTUs with an adjusted *P*-value <.05 and a > 2-fold change were considered differentially abundant in response to the fertilizer regime. OTUs with significantly higher abundance under the compost regime were designated as compost-sensitive taxa; those with significantly higher abundance under the digestate regime were designated as digestate-sensitive taxa; and OTUs that were significantly more abundant under the reference regime—i.e. less abundant under compost or digestate—were designated as reference regime-specific taxa. Differentially abundant OTUs were further tested for their linear relationship with the quantity of compost or digestate applied to the field. To distinguish persistent compost-associated taxa from transient responders to the most recent amendment, the detrended relative abundance of each compost-sensitive OTU was additionally regressed against the interval between the last fertilization event and soil sampling, with the quantity of compost applied included as a covariate. To test for enrichment of bacterial or fungal taxa (e.g. phyla) or functional guilds, we constructed a contingency table for each taxon or guild testing whether a given taxonomic group was over-represented among compost-, digestate-, or reference-sensitive OTUs relative to the full OTU pool, and tested for significance with Fisher’s exact test. Phyla, orders, families, and genera with an adjusted *P*-value (FDR) below .05 were considered significantly enriched.

#### Microbial co-occurrence networks

OTUs present in fewer than 20% of samples were excluded prior to network construction. We constructed a co-occurrence network based on a combined bacterial and fungal OTU table, utilizing the PLNnetwork function in the R package PLNmodels and employing a sparse multivariate Poisson log-normal (PLN) model [36], following the Extended Bayesian Information Criterion (EBIC) as performed earlier [37]. Unlike univariate correlation-based methods, PLNmodels accounts for compositionality while controlling for technical and environmental variation. The model included MAP, MAT, altitude, sand, and clay as covariates and the logarithm of sequencing depth as an offset. To define modules and network node characteristics, we calculated ∣z∣ (within-module degree) and P (participation coefficient) to identify peripheral and hub taxa within the network [38], using the packages RNetCarto [39] and igraph [40], retaining only modules with >10 nodes. The relative abundance of each module was calculated as the summed relative abundance of all OTUs assigned to that module within each sample’s subnetwork [37]. Using the network meta-matrix, each sample’s network was subset to include only detected OTUs [41]. For each subnetwork, links were counted, and complexity was determined as linkage complexity (links per taxon, [42]) for bacterial, fungal, and bacterial–fungal links. Differential abundance analysis and network construction were performed independently; compost-sensitive OTUs identified through the former were subsequently mapped onto the network to examine their module membership.

#### Direct and indirect relationships of microbial community traits with proxies for soil functions

We first tested relationships between microbial community traits (diversity, structure, network complexity) and two soil function proxies—basal respiration and aggregate stability [43], using Spearman correlations with FDR correction in the microbiome package [44].

To disentangle direct and indirect effects of compost application, pedoclimatic factors (texture, MAT, MAP), soil physicochemical properties (SOC, pH, bulk density), and microbial traits on soil functions, we built structural equation models (SEMs) in lavaan [45]. An a priori path diagram (Fig. S2) was constructed based on established scientific evidence, enabling us to assess how compost application and inherent pedoclimatic factors (soil texture, MAT, MAP) relate to manageable soil physicochemical properties (SOC, pH, bulk density), microbial community traits, and ultimately soil functions [46]. We ran separate SEMs for bacterial traits, fungal traits, and microbial modules to simplify pathways. Model fit was assessed by χ^2^  *P* > .05, CFI > 0.90, TLI > 1, and RMSEA <0.08, RMSEA *P* > .05 using semTools [47].

## Results

### Microbial diversity and community structure

Soil physicochemical, spatial, and climatic factors explained most of the variation in both bacterial and fungal community composition. For bacteria, these environmental predictors accounted for the largest share of variation (partial *R*^2^ = 0.462), with hierarchical modelling identifying soil pH, clay content, and bulk density as the strongest individual predictors (Fig. 1A; Table S1). Management effects were much smaller (management system: partial *R*^2^ = 0.034; fertilizer regime: partial *R*^2^ = 0.067), although ordination analyses still showed weak but consistent patterns (Fig. 1A, Fig. S3A). Pairwise comparisons among management systems and fertilizer regimes were not significant, and multivariate dispersion tests confirmed that beta-diversity differences were not caused by within-group variability.

**Figure 1:**
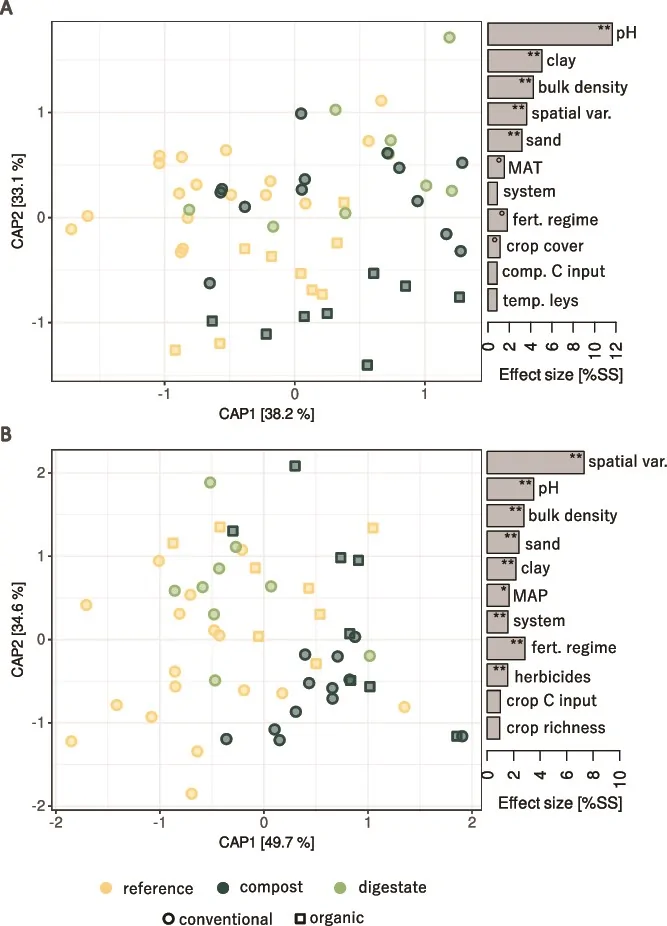
Effects of management and environmental factors on bacterial and fungal community structures. Ordination plots display the CAP for soil bacterial (A) and fungal (B) communities, constrained by management systems (conventional and organic) and fertilizer regimes (reference, compost, and digestate). For bacterial communities (A), pairwise comparisons of community structure among fertilizer regimes and management systems were not significant. For fungal communities (B), pairwise comparisons of community structure revealed significant differences among fertilizer regimes (reference vs. compost: *P* < .001; reference vs. digestate: *P* = .11) and between management systems (*P* = .006). Adjacent bar plots show the effect sizes of primary environmental and management factors, expressed as the percentage of the total sum of squares (SS), based on PERMANOVA analysis. Asterisks indicate significance levels (° *P* < .1; ^*^  *P* < .05, ^**^  *P* < .01, ^***^  *P* < .001).

For fungi, variance decomposition similarly attributed most variation to environmental factors (partial *R*^2^ = 0.401), with hierarchical modelling identifying spatial eigenvectors, pH, bulk density, texture, and precipitation as the main individual predictors (Fig. 1B; Table S1). However, fungal communities responded more strongly to management than bacterial communities. The fertilizer regime showed the clearest effect (partial *R*^2^ = 0.091), followed by the management system (partial *R*^2^ = 0.055). Ordination analyses confirmed distinct management-related patterns (Fig. 1B, Fig. S3B), and pairwise tests showed significant differences among fertilizer regimes (reference vs. compost*: P <* .001; reference vs. digestate: *P =* .11) and among management systems (*P* = .006). Herbicide use also affected fungal composition, although the effect was small (Fig. 1B; Table S1). No treatment-related dispersion effects were detected.

Bacterial diversity metrics (Shannon diversity and evenness) were primarily explained by soil physicochemical, spatial, and climatic factors. Variation partitioning assigned most explained variance to these environmental predictors in both Shannon diversity (partial *R*^2^ = 0.3) and evenness (partial *R*^2^ = 0.439), with hierarchical modelling identifying bulk density and sand content as the main individual predictors (Figs 2A and S4A, S4C; Table S2). Management effects were limited: the management system (partial *R*^2^ = 0.084) and fertilizer regime (partial *R*^2^ = 0.008 for Shannon; 0.037 for evenness) contributed only modestly, although both indices tended to be slightly higher under organic management. Crop cover and herbicide use were the strongest management-related individual predictors, showing positive and negative associations, respectively (Table S2).

**Figure 2:**
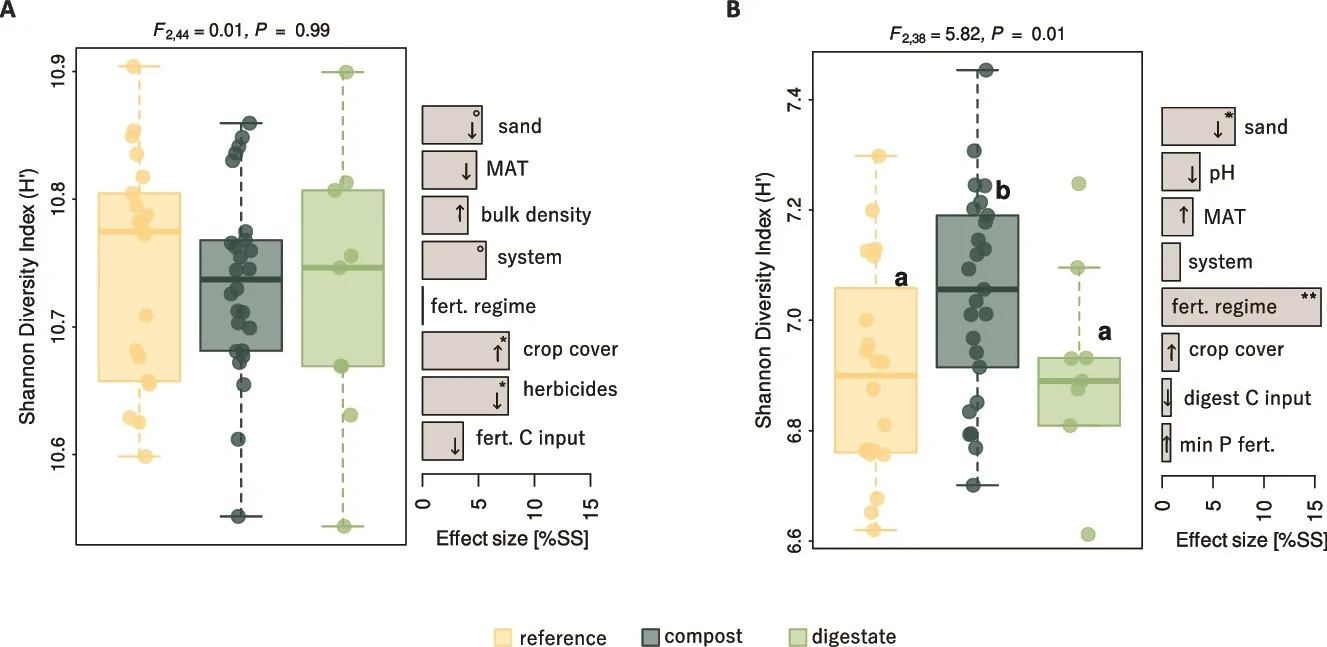
Bacterial and fungal diversity as influenced by management and pedoclimatic conditions. Box plots illustrate the effect of fertilizer regimes (reference, compost, and digestate) on soil bacterial (A) and fungal (B) diversity, as measured by the Shannon–Wiener diversity index. The F-statistic and corresponding p-value from the linear model are presented above each box, indicating the statistical significance of the effect of the fertilizer regime on diversity. Different letters above the boxplots denote significant differences between the fertilizer regimes. Adjacent bar plots illustrate the primary pedoclimatic and management drivers of bacterial and fungal diversity, respectively, as percentages of the total sum of squares (SS). The direction of the effect, whether positive or negative, is indicated with arrows. Significance levels are indicated by stars (° *P* < .1; ^*^  *P* < .05, ^**^  *P* < .01, ^***^  *P* < .001).

Fungal diversity showed a stronger response to management, particularly to the fertilizer regime. While variation partitioning attributed most explained variance to environmental factors for both Shannon diversity (partial *R*^2^ = 0.375) and evenness (partial *R*^2^ = 0.468), mainly driven by sand content, pH, and precipitation (Fig. 2B; Table S3), the fertilizer regime explained substantial additional variation (partial *R*^2^ = 0.22 for Shannon; 0.118 for evenness). Compost-amended soils showed the highest fungal Shannon diversity and tended to exhibit higher evenness (Fig. 2B, Fig. S4B, Fig. S5B). The management system contributed only marginally to both indices (partial *R*^2^ < 0.01), and no other individual management factors emerged as consistent predictors in the hierarchical modelling (Table S3).

#### Taxonomic composition and fertilizer-sensitive taxa

Amplification of 16S rRNA and ITS gene fragments resulted in 6748 bacterial and 4105 fungal OTUs. After filtering low-abundance OTUs, 3038 bacterial and 660 fungal OTUs remained for analysis. The bacterial community was dominated by Proteobacteria, Actinobacteria, and Acidobacteria (>74% combined), with Chloroflexi, Bacteroidetes, Verrucomicrobia, and Firmicutes as subdominant phyla (Fig. S6A). Fungal communities were dominated by Ascomycota (48.7%) and Mortierellomycota (29.5%), followed by Basidiomycota (6.6%; Fig. S6B).

We identified OTUs that responded sensitively to specific fertilizer regimes by testing for differential abundance after correcting for pedoclimatic conditions, as previously conducted [48]. The fertilizer-sensitive taxa were primarily recorded under the compost fertilizer regime, for both bacteria (*n* = 13; Fig. S7) and fungi (*n* = 9; Fig. S8), with five bacteria and four fungi also enriched in the digestate regime. Only one fungal taxon, but no bacterial taxa, was exclusively enriched in the digestate regime, while we identified several OTUs that were more prevalent under the reference fertilizer regime (Figs S7 and S8). The compost-sensitive OTUs (i.e. differentially abundant under the compost regime, as defined in the Methods) showed predominantly positive correlations with the amount of compost applied, particularly for bacteria (Figs S9 and S10). Conversely, for OTUs enriched under the digestate regime, a clear positive relationship was observed only for one fungal taxon. Compost fields were sampled between 158 and 1430 days after the last fertilization event (median 331 days). None of the 13 bacterial or nine fungal compost-sensitive OTUs showed a significant association between abundance and this interval; one unclassified Actinomycetales OTU showed a tendency to decline (bOTU52707, *P* = .08; Figs S11 and S12). The interval slope was unrelated to OTU prevalence in either domain (Spearman rho = 0.22, *P* = .46 for bacteria; rho = 0.43, *P* = .25 for fungi*).*

The bacterial fertilizer-sensitive taxa belonged to several phyla, mainly Actinobacteria, Firmicutes, and Proteobacteria (Table S4). Under compost, Firmicutes, along with the class Bacilli and the family Bacillales, were significantly enriched, whereas under digestate, only the phylum Firmicutes showed significant enrichment (Table S5). The bacterial compost-sensitive taxa harboured some thermophilic families, such as Thermomonosporaceae and Thermoactinomycetaceae. Most fungal fertilizer-sensitive taxa belonged to the phylum Ascomycota, with the predominant class being Sordariomycetes (Table S6). Notably, one compost-sensitive OTU was classified as Thermomyces, another thermophilic genus. However, no taxonomic groups were significantly enriched in the fertilizer-sensitive fungi. We also evaluated different trophic guilds of bacteria and fungi, but none emerged as particularly enriched among the taxa sensitive to the fertilizer regime.

#### Co-occurrence network properties

The co-occurrence network consisted of 214 nodes and 907 edges (*P* < 0.05; associations between taxa). Nodes averaged 8.48 connections (max = 129), with a clustering coefficient of 0.23 and modularity of 0.37. It was clustered into four top modules with varying characteristics and responses to fertilizer regimes (Fig. 3A).

**Figure 3:**
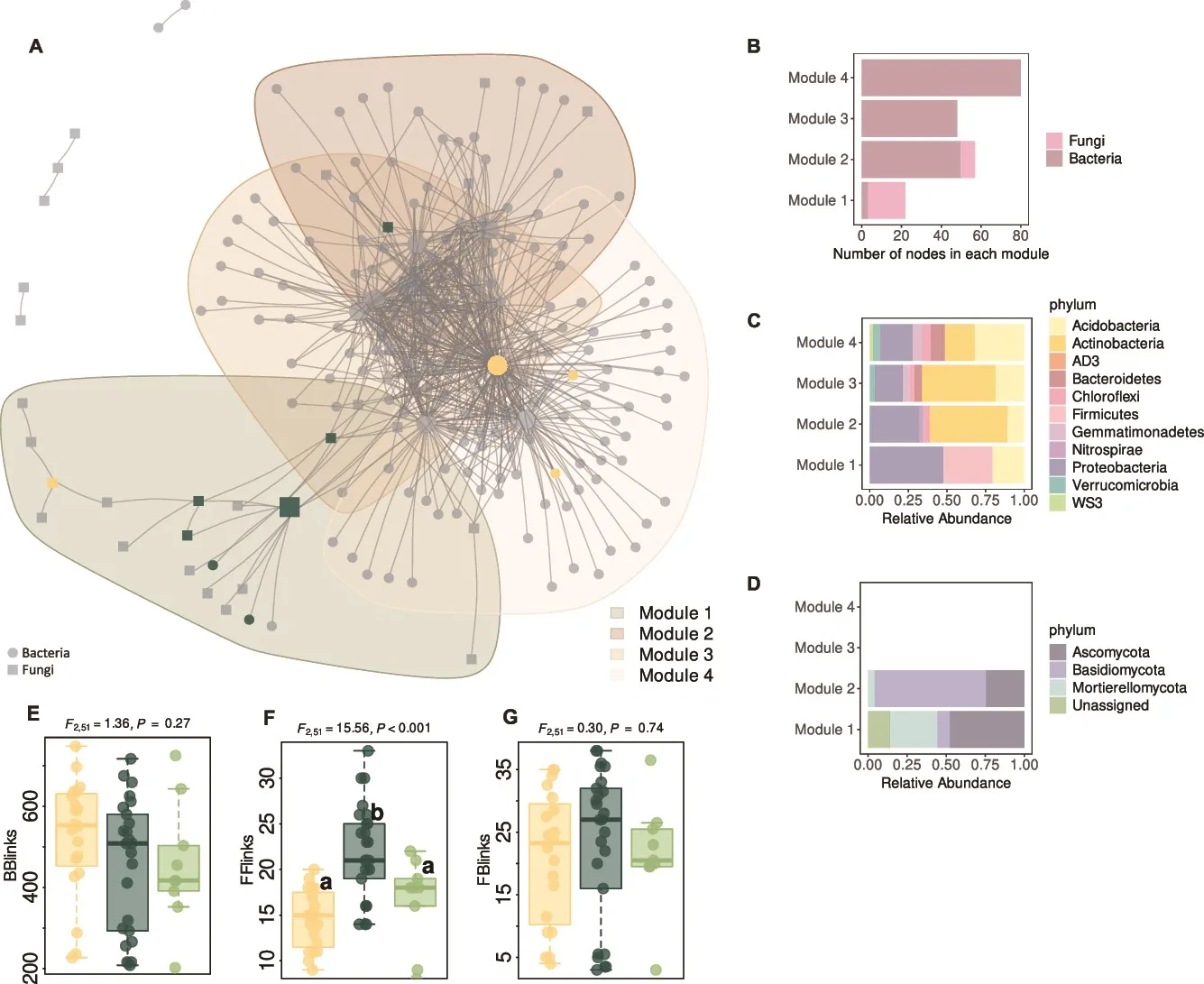
Microbial co-occurrence network and module composition. (A) Network inferred from bacterial and fungal OTU tables across all samples. Node colour marks taxa sensitive to the fertilizer regime, and node size denotes topological role (peripheral to connector hub). Shading indicates affiliation with the four largest modules. (B) Number of nodes per module, split by bacteria and fungi. (C–D) Phylum-level taxonomic composition within each module for bacterial (C) and fungal (D) taxa, shown as relative abundance. (E–G) Network complexity of bacterial (BB), fungal (FF), and fungal–bacteria (FB) associations under each fertilizer regime; different letters denote significant pairwise differences (*P* < .05).

Module 1, the smallest module, comprised only 22 nodes and was uniquely dominated by fungal taxa (Fig. 3B). This module harboured the highest number of compost-sensitive taxa (*n* = 5), with a higher relative abundance under frequent compost application, although not statistically significant (Fig. S13A), and showed almost only positive associations (Fig. S13E). In contrast, module 4 was the most extensive, comprising over 80 nodes that consisted solely of bacterial taxa (Fig. 3B). The relative abundance of module 4 tended to be highest in soils receiving frequent digestate applications (Fig. S13D). Module 2 was also larger, with a clear contribution from fungal taxa, and showed the highest negative association proportion (Fig. S13E). The taxonomic composition of the modules varied markedly. Module 1 was dominated by bacterial phyla Proteobacteria and Firmicutes—the latter of which were nearly absent in the other modules—as well as the fungal phylum Ascomycota (Fig. 3C and D). In contrast, the remaining modules showed higher abundances of Actinobacteria and Bacteroidetes and were primarily dominated by the fungal phylum Basidiomycota.

To explore the effect of fertilizer regimes on microbial network complexity, we compared the number of associations within the co-occurrence network across regimes. Under frequent compost application, fungal–fungal associations were notably higher (Fig. 3F). In contrast, bacterial and bacterial-fungal association complexity showed no consistent differences among fertilizer regimes (Fig. 3E and G).

#### Microbial community traits and their direct and indirect link to soil functions

Spearman correlations revealed no clear association between microbial diversity or network complexity and soil functions, whereas bacterial community composition was strongly linked to soil respiration and fungal community composition was associated with both soil respiration and aggregate stability (Fig. S14). The relative abundances of modules 2 and 4 were additionally negatively correlated with soil respiration.

Contrary to the univariate associations, SEMs suggested that microbial community traits had stronger direct associations with aggregate stability than with basal respiration (Fig. 4). Fungal diversity and the relative abundance of module 4 were positively and negatively associated with soil respiration, respectively, whereas bacterial diversity, fungal community composition and association complexity, and modules 1–3 were all positively associated with aggregate stability (Tables S7–S9). Soil functional proxies were also strongly associated with physicochemical properties: basal respiration was mainly associated with SOC, while aggregate stability was primarily associated with bulk density. Microbial traits covaried strongly with soil physicochemical conditions—particularly community composition, association complexity, and module abundances—whereas diversity indices showed weaker covariation (Tables S7–S9). The SEMs indicated that compost was linked to soil functions predominantly through indirect pathways (Fig. 4B and C). Compost was associated with higher soil respiration, with the pathway running primarily through increased SOC and to a smaller extent, via higher fungal diversity. The influence of compost on aggregate stability was less clear, showing on the one hand a positive pathway through fungal community composition and association complexity, but on the other hand a negative indirect pathway via compost-induced increases in soil pH.

**Figure 4:**
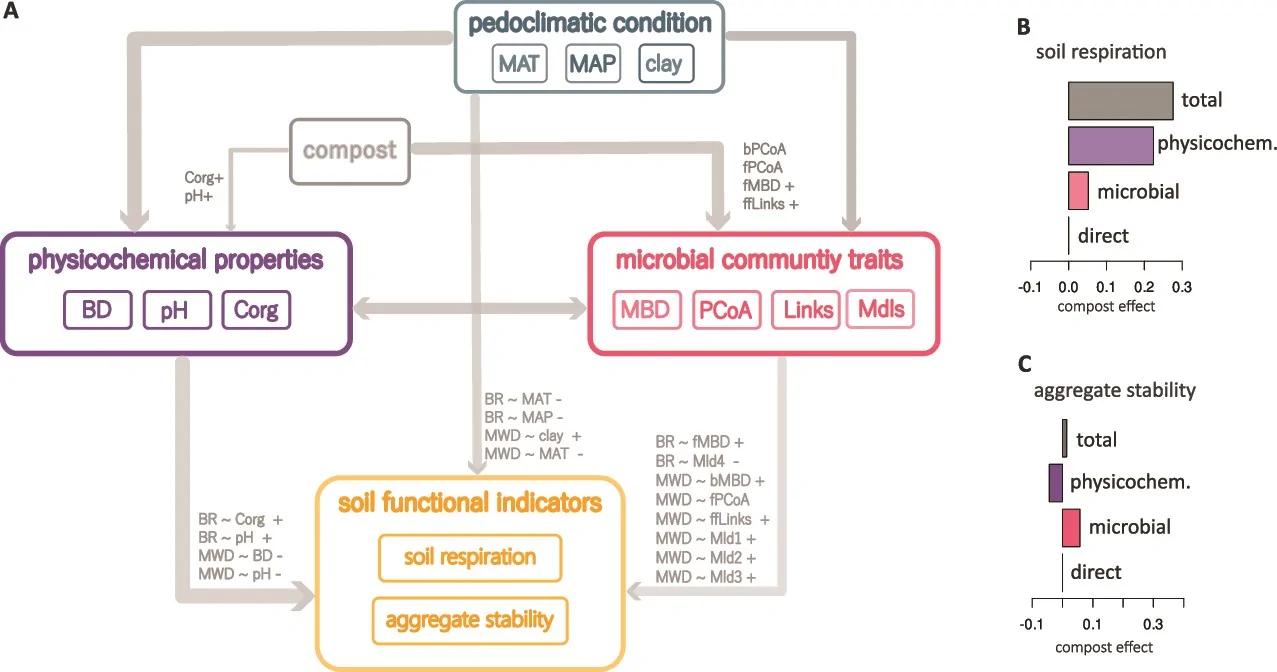
Direct and indirect effects of compost on soil functional indicators. (A) Structural equation models (SEMs) show associations between pedoclimatic conditions, compost application, soil physicochemical properties, and microbial community traits, with soil respiration and aggregate stability. Separate SEMs were built for bacterial, fungal, and microbial module traits. Line thickness represents the absolute value of the standardized coefficients (scaled to the highest coefficient within each group). A “+” or “–” after each variable indicates the direction of the standardized coefficient (except for associations with PCoA axes, which are not directional). A prefix b or f indicates bacterial or fungal traits. (B–C) Bar plots showing the direct and net indirect effects of compost on soil respiration (B) and aggregate stability (C). Net indirect effects represent the sum of indirect pathways through microbial traits or soil physicochemical properties. Exact standardized coefficients, standard errors, significance values, and *R*^2^ values for all SEMs are provided in Tables S7–S9. Variable abbreviations: BR = soil respiration, MWD = index for aggregate stability, BD = bulk density, MAT = mean annual temperature, MAP = mean annual precipitation, C_org = soil organic carbon, MBD = microbial biodiversity (Shannon index), PCoA = index for microbial community structure, links = association complexity, and Mdls = microbial module indicators. A prefix b or f indicates bacterial or fungal traits.

## Discussion

The use of compost is widely acknowledged as an effective approach to enhancing agricultural soil health, particularly by enriching and stimulating the soil microbial community. However, the existing evidence largely stems from field experiments that apply specific composts and are conducted under a singular set of pedoclimatic conditions [15, 18]. Here, we overcome this limitation by investigating the effects of compost application on a network of 56 farms employing different fertilization regimes—including regular application of compost from diverse sources—across a broad range of management and pedoclimatic conditions. Our results suggest that compost is associated with consistent shifts in soil fungal community composition possibly by introducing new taxa that establish a distinct cluster within the soil microbial community.

### Compost application shapes diversity, composition, and complexity of soil fungal communities

Compost consistently enhanced fungal diversity, shifted fungal community composition, and markedly increased fungal network complexity. These three complementary lines of evidence indicate a consistent compost-associated signature in the soil fungal community, which exceeded the impact of other management factors and even that of the overall management system (organic versus conventional). Compost effects on soil fungal communities have been reported previously—compost has been shown to increase fungal diversity and shift community composition [49, 50] and to enhance fungal network complexity in particular [50–52].

The soil bacterial community, in contrast, was less clearly affected by the fertilizer regimes—a pattern less consistent with the broader literature, where only a few studies report stronger fungal than bacterial responses [53, 54], but most indicate comparable effects on both groups [6, 15, 17, 50, 55], or stronger bacterial responses [18, 56]. A key distinction in our study is that fertilizer regimes differed only marginally in carbon input (see Supplementary Results); notably, the reference regime also received substantial organic carbon (e.g. through manure), unlike most studies comparing compost to mineral fertilizer-only or unfertilized controls [6, 15, 18, 49, 50, 53–56]. The fact that a consistent fungal community signature remains detectable against this more realistic comparison highlights the specificity of the compost effect on fungal communities. The stronger bacterial responses reported in earlier studies may therefore partly reflect the large contrast in total carbon inputs between treatments rather than a specific effect of compost per se. This suggests that fungal communities are sensitive to the qualitative characteristics of the organic amendment, whereas bacterial communities appear to respond primarily to the magnitude of organic carbon input. Such a pattern is consistent with the known niche differentiation between fungi and bacteria—fungi are inherently more competitive in utilizing structurally complex and recalcitrant organic substrates [57, 58]. Compost, as a humified and microbially processed amendment, provides a more recalcitrant substrate than the fresh manure received with regular manure or slurry application as for the reference treatment in our study [49, 59, 60], making it particularly well-suited to stimulate fungal over bacterial communities.

At the individual taxon level, however, a comparable number of bacterial and fungal taxa responded to the different fertilizer regimes. Many of the compost-responsive taxa were characteristic of thermophilic groups typically enriched during composting processes, such as *Thermomonospora* and *Thermomyces* [61]. Notably, members of the phylum Firmicutes, particularly the class Bacilli, were strongly enriched among compost-sensitive taxa, aligning with their well-documented prevalence in compost materials and compost-amended soils [15, 61, 62]. The convergence of multiple independently identified OTUs on thermophilic lineages, alongside consistent Firmicutes enrichment, provides biological validation beyond statistical inference alone, suggesting these responses reflect a compost-associated signal rather than random microbiota shifts.

A notable proportion of the compost-sensitive taxa clustered within a small but distinct cluster (Module 1), dominated overall by fungal taxa but containing a notably high proportion of the compost-associated bacterial phylum Firmicutes. Module 1 was also particularly interesting as it displayed the highest proportion of positive associations—indeed, compost-sensitive taxa within this module were connected solely by positive links. This strong prevalence of positive associations reflects earlier findings showing that compost application is associated with positive links in microbial co-occurrence networks [51, 52]. The combination of fungal dominance, high Firmicutes prevalence, clustering of compost-sensitive taxa, and exclusively positive internal associations suggests that Module 1 may represent a distinct cluster with a partial compost-derived origin, even though its overall abundance did not differ significantly between fertilizer regimes. Such formation of distinct microbial clusters following compost addition has also been reported previously [16], consistent with a community coalescence framework, in which the compost-borne microbiome partially establishes within the resident soil community [19]. Nevertheless, based on the current data, we cannot determine whether the compost-responsive taxa originated from the compost itself or whether they were resident soil microbes stimulated by the compost addition.

Previous studies have mostly examined compost effects on soil microbial communities in single fields and tracked only short-term or intermediate responses [4, 6, 15, 18]. In contrast, our study provides a broader perspective by assessing compost-related microbial responses across 56 fields with varying compost sources and management histories—spanning both organic and conventional farms across gradients of soil type and climate within Swiss temperate agroecosystems. A consistent compost signature remained detectable across this heterogeneous network. Notably, the abundance of compost-sensitive OTUs was unrelated to the interval since the last amendment, which ranged from five months to nearly four years. This argues against transient stimulation by the most recent application and instead indicates establishment within the resident soil community. However, the magnitude of these effects likely varies across contexts, as pedoclimatic conditions and management history shape the resident community [63, 64] and earlier work showed that the initial microbial community composition was a stronger predictor of compost taxon establishment than management system alone [65]. Compost effects may also be more pronounced in conventionally managed soils due to the stronger contrast with a low organic input history [65–67]. Whether equivalent patterns emerge under contrasting climatic or cropping contexts remains an open question for future work.

### Subtle modulation of the soil microbial community by solid digestate

In contrast to compost, the application of digestate induced more subtle changes in the soil microbial community. Indeed, microbial community traits in soils under regular digestate application were often indistinguishable from those in the reference treatment and overlapped with those under the compost regime. Similarly, many taxa sensitive to compost were also enriched under frequent digestate application, and only one fungal taxon was found to be exclusively responsive to digestate. This outcome is somewhat unexpected, as digestates have previously been shown to affect soil microbial communities and specifically introduced some rare phyla such as Chloroflexi [68, 69]. Indeed, we also observed higher abundances of Chloroflexi in Module 4, which tended to be more abundant under digestate treatment. However, this pattern was not statistically significant and should therefore be interpreted with caution. Remarkably, in one of the earlier studies [68], the effect of digestate was highly soil-type dependent and may have been masked across the wide range of soil textures explored in the current study. Moreover, the weaker digestate signal may partly reflect limited statistical power (*n* = 9 vs. *n* = 26 for compost), warranting further investigation.

### Impact of management on soil microbial communities beyond fertilization regimes

Our findings indicated that while the fertilizer regime primarily influenced the soil fungal community, the bacterial community was more affected by other management factors such as management system or factors related to crop rotation and pesticide use. Bacterial diversity indices showed a weak response to the management system, with models suggesting a marginal increase under organic management. This observation aligns with previous research indicating that organic farming enhances soil microbial diversity and richness compared to conventional agriculture [67, 70, 71]. A key feature of organic agriculture is the reduction or elimination of pesticide use, which is known to strongly influence soil microbial communities [48, 72]. In our study, herbicide use was negatively associated with bacterial diversity, suggesting that lower herbicide inputs may be one of the factors contributing to the higher microbial diversity often observed in organic compared with conventional systems. Another strong management factor was crop cover, which has already been reported as an important driver of soil microbial communities [73, 74]. Notably, when comparing management factors across the three fertilizer regimes, only practices directly linked to fertilizer (e.g. organic C input, mineral N input) differed consistently between the regimes. Other management factors—such as crop cover, tillage, and pesticide use—did not show systematic differences between the regimes (see Supplementary Results). The observed microbial differences were thus primarily tied to fertilizer regime, though practices reducing chemical inputs or enhancing soil cover likely play a complementary role.

### The role of soil microbiota in the different pathways of compost in fostering soil functions

While our study shows clear effects of compost on soil microbial communities, its broader agroecological significance lies in its impact on soil health—encompassing not only microbial community traits but also their links to soil physicochemical properties and ultimately soil functioning. Such benefits of compost application are well established [3–5] and consistent with earlier work on the same network [7]. That study showed that compost application improved overall soil health across this farm network—notably driven by higher soil respiration and cation exchange capacity, and partly by a trend towards increased carbon stocks—with no yield penalty observed. The beneficial effects of compost on soil health are often strongly associated with increased microbial diversity and activity [6, 7, 16]. However, since compost also alters soil physicochemical properties, it remains unclear whether the observed effects are primarily driven by microbial changes [17].

To explore this, we employed SEMs that combined microbial community traits, soil physicochemical properties, and soil functional proxies. The association between compost and soil functional proxies was most evident for soil respiration, where it was primarily mediated through increases in soil organic carbon. This aligns with the well-established role of SOC as a major driver of microbial activity [75, 76]. Although compost is also expected to enhance soil respiration through direct mechanisms—such as the provision of readily available nutrients and labile carbon that stimulate microbial metabolism [77, 78]—such direct associations were not evident in our SEMs. Indirect associations mediated through microbial community traits were weaker but detectable, particularly via fungal diversity. This aligns with earlier findings reporting associations between fungal richness and soil respiration [79, 80]. In contrast to soil respiration, compost was associated with higher aggregate stability with microbial community traits contributing positively to this pattern through fungal community composition and association complexity, consistent with the well-established role of microbial communities in soil structure formation and stabilization [10, 81]. Such indirect compost effects remain easily hidden when microbial pathways are not explicitly integrated into soil functional assessments [7]. Beyond microbial influences, the SEMs highlighted that soil functional proxies were strongly shaped by physicochemical properties—most notably SOC and bulk density—underscoring the importance of favourable soil conditions that support, but also extend beyond, microbial community functioning [82, 83]. Moreover, microbial traits were tightly linked to these soil properties, illustrating the fundamental interplay between microbial communities and the chemical and physical characteristics of soils [76]. It is essential to note that SEMs explore possible pathways, rely solely on correlations, and cannot establish definitive causation. Controlled experiments, such as comparing sterilized and non-sterilized compost, are needed to deepen our mechanistic understanding of the manifold effects of compost on soil functions. Nonetheless, our on-farm observational study provides field-based evidence of compost’s potential to enhance soil functions through multiple pathways across diverse conditions.

Our study indicates that compost application is consistently associated with enhanced fungal diversity, increased fungal network complexity, and the formation of a distinct fungus-rich microbial module—community-level changes that are in turn linked to key soil functional proxies including basal respiration and aggregate stability. These findings underline compost’s capacity to strengthen multiple aspects of soil health and support more resilient agricultural systems. However, compost is not a stand-alone solution and must be integrated thoughtfully into broader sustainable management strategies. A central limitation is its constrained availability. Compost production typically relies on organic materials sourced from other locations, which may lead to carbon and nutrient depletion at donor sites—an issue that applies to most organic amendments [84]. Thus, compost use is most sustainable when viewed within a landscape-level circularity framework that balances inputs and outputs across the system. Another concern is the risk of contamination with plastics and other hazardous materials, which can diminish the positive impacts of compost on soil microbial communities, soil functions, and ultimately primary productivity [85–87]. Despite these challenges, our findings reinforce the role of composting as a cornerstone practice in sustainable soil management—both as a means of recycling organic waste and as a strategy to enhance soil functioning through improved microbial dynamics. When applied within a well-designed management regime, compost represents a valuable component of sustainable agricultural soil stewardship.

## Supplementary Material

Supplementary_Information_final_ycag259

## Data Availability

Amplicon sequencing data are publicly available in the NCBI Sequence Read Archive (SRA) under BioProject accession PRJNA1440939.
