# Supplementary material for: Compost application more strongly shapes soil fungal than bacterial communities across arable fields
Source: ISME Commun. 2026 Sep 10;6(1):ycag259. doi: 10.1093/ismeco/ycag259 (PMC13657048; doi:10.1093/ismeco/ycag259)
Supplement: Supplementary file 1 [file supplementary_information_final_ycag259.pdf]

Supplementary Information: Compost application more strongly shapes soil fungal than bacterial communities across arable fields

Florian Walder, Kyle Hartman, Anna Edlinger, Chantal Herzog, Alain Y. Held, Sana Romdhane, Laurent Philippot, Samiran Banerjee and Marcel van der Heijden

Supplementary Materials and Methods

Microbial Community Profiling

The assessment of prokaryotic and fungal communities was carried out using sequencing methods outlined in [1] and [2]. To summarise the key steps, DNA was extracted from the frozen composite soil samples using the DNeasy PowerSoil-htp 96 well DNA isolation kit following the manufacturer's protocol (Qiagen, France). DNA concentrations were measured using a Qubit fluorometer (Life Technologies, Paisley, UK).

The prokaryotic communities were assessed by amplifying the V3-V4 region of the 16S rRNA gene using the primers 341F (CCTACGGGNGGCWGCAG) and 805R (GACTACHVGGGTATCTAATCC) [3]. Subsequently, Illumina 300 bp paired-end sequencing was performed at the Functional Genomic Centre Zurich (FGCZ, Zurich, Switzerland http://www.fgcz.ch). The bioinformatics process involved quality checking of R1 and R2 reads using FastQC [4] and trimming. The trimmed reads were then merged, allowing a minimum overlap of 15 bp using FLASH v1.2.11 [5]. FASTA-formatted sequences were extracted from FASTQ files and quality-filtered using PRINSEQ-lite v0.20.4 [6]. The filtering parameters included a GC range of 30-70, a minimum mean quality score of 20, no ambiguous nucleotides, and a low-sequence-complexity filter in the DUST algorithm with a threshold of 30. The reads were demultiplexed using an in-silico PCR approach in USEARCH (v11), allowing up to 1 mismatch in the barcode-primer sequence but not at the 3-prime end. Subsequently, the sequences were clustered into operational taxonomic units (OTUs) based on 97% similarity, using the UPARSE pipeline [7]. Taxonomical information was predicted for bacterial OTUs based on the SILVA v128 database [8], and any non-bacterial OTUs were excluded. To minimise noise from low-abundance and potentially spurious OTUs, we retained only those with a total read count of at least 50 across all samples and that were present in at least 5 samples.

The fungal ITS region was amplified using the primers ITS1F (CTTGGTCATTTAGAGGAAGTAA) and ITS4 (TCCTCCGCTTATTGATATGC) to target the entire ITS region [9]. Subsequently, the amplified region was sequenced on the PacBio® Sequel Instrument using the blunt-end ligation protocol of PacBio (2kb library preparation; Pacific Biosciences, CA) at FGCZ, Zurich. The sequencing libraries were prepared using P6/C4 chemistry (DNA/Polymerase Binding Kit P6, DNA Sequencing Reagent 4.0). The raw sequences were processed using the PacBio SMRT Portal (https://www.pacb.com/products-and-

services/analytical-software/smrt-analysis/) to extract circular consensus sequences, with a minimum of 5 passes. The sequencing data underwent quality filtering using PRINSEQ-lite v0.20.4 [6], with the following filtering parameters: GC range of 30-70, minimum mean quality score of 20, absence of ambiguous nucleotides, and a low-complexity filter in the DUST algorithm with a threshold of 30. Subsequently, read demultiplexing was carried out using an in-silico PCR approach as part of USEARCH v11 [10], allowing up to 1 mismatch in the barcode-primer sequence, excluding mismatches at the 3-prime ends. The amplicon size range was defined as 100 – 2000, and any amplicons with additional primer sites (concatenated amplicons - multi-primer artefacts) were eliminated. Following this, the sequences were clustered into operational taxonomic units (OTUs) at a 97% similarity threshold using the UPARSE pipeline [7]. Taxonomic information was assigned to the OTUs using the UNITE database (V7.2) [11] and SINTAX. The taxonomic predictions were validated using ITSx [12], and any non-fungal OTUs were excluded. To minimise noise from low-abundance and potentially spurious OTUs, we retained only those OTUs with a total read count of at least 5 across all samples and with presence in at least 5 samples.

Putative functions were inferred from taxonomy. Bacterial OTUs were annotated with FAPROTAX [13]; fungal OTUs were assigned guilds with FUNGuild [14]. For bacteria, FAPROTAX functions were collapsed into six broad modes: Chemoheterotrophs (chemoheterotrophy, aerobic_chemoheterotrophy, anaerobic_chemoheterotrophy), Nitrifiers (nitrification, ammonia_oxidation), Denitrifiers (nitrate_reduction, nitrite_reduction, denitrification), Nitrogen fixers (nitrogen_fixation), Sulfur cyclers (sulfate_reduction, sulfur_oxidation), and Human pathogens (human_pathogens_all, human_pathogens_pneumonia) following Labouyrie et al [15]. Fungal trophic modes followed FUNGuild assignments. Taxa without a match were kept as unknown. These annotations are predictions based on taxonomy and were used for comparative (not absolute) interpretations.

Supplementary Results

Management Background

In addition to the management system and fertiliser regime, we evaluated several other management variables across the 56 study fields. We detected significant differences among fertiliser regimes only for fertilisation-related inputs (Fig. S15). The reference group received the highest manure input, although all fields received some manure; in contrast, the compost and digestate regimes received either compost or digestate exclusively. Total organic-fertiliser carbon input differed between fertiliser regimes: compost-treated fields received the highest inputs (mean = 10.6 ± 5.7 t C ha^{-1}), followed by digestate fields (mean = 9.2 ± 2.8 t C ha^{-1}), and reference fields the lowest (mean = 6.4 ± 2.7 t C ha^{-1}). Conversely, the mineral Nitrogen fertiliser input (including the mineral fraction of organic amendments) was highest in the reference fields and lowest under compost treatment, with the digestate fields again being intermediate. All other variables – such as fungicide and herbicide intensity, tillage frequency, crop species richness - did not differ among fertiliser regimes. However, fields under regular digestate application tended to have a lower proportion of leys.

Supplementary Figures

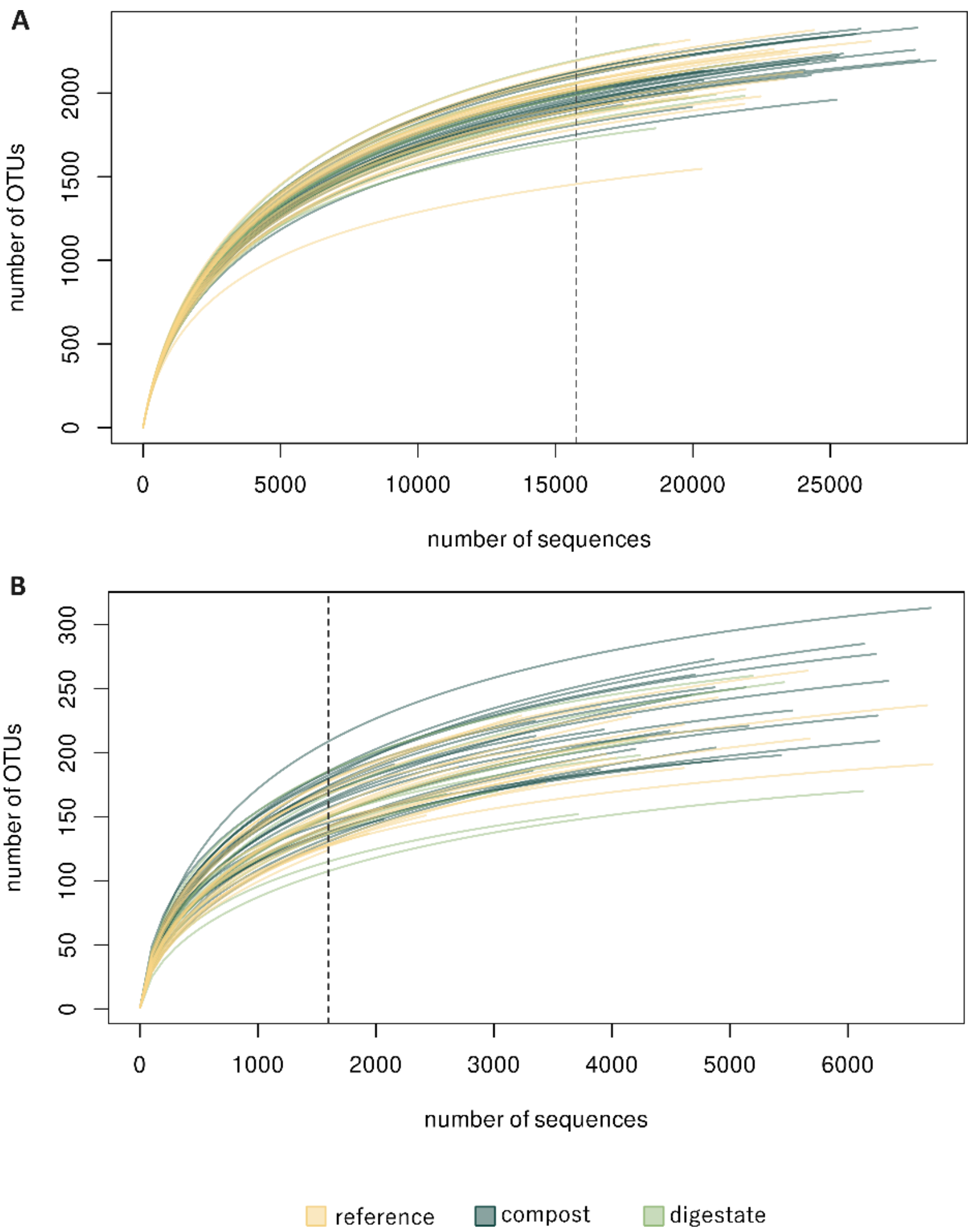

Figure S1 Rarefaction curves of bacterial (A) and fungal (B) communities. The curves are coloured by fertiliser regimes, with the black horizontal lines indicating the sampling depths used for the rarefaction analysis (15'759 reads for bacteria; 1`597 for fungi).

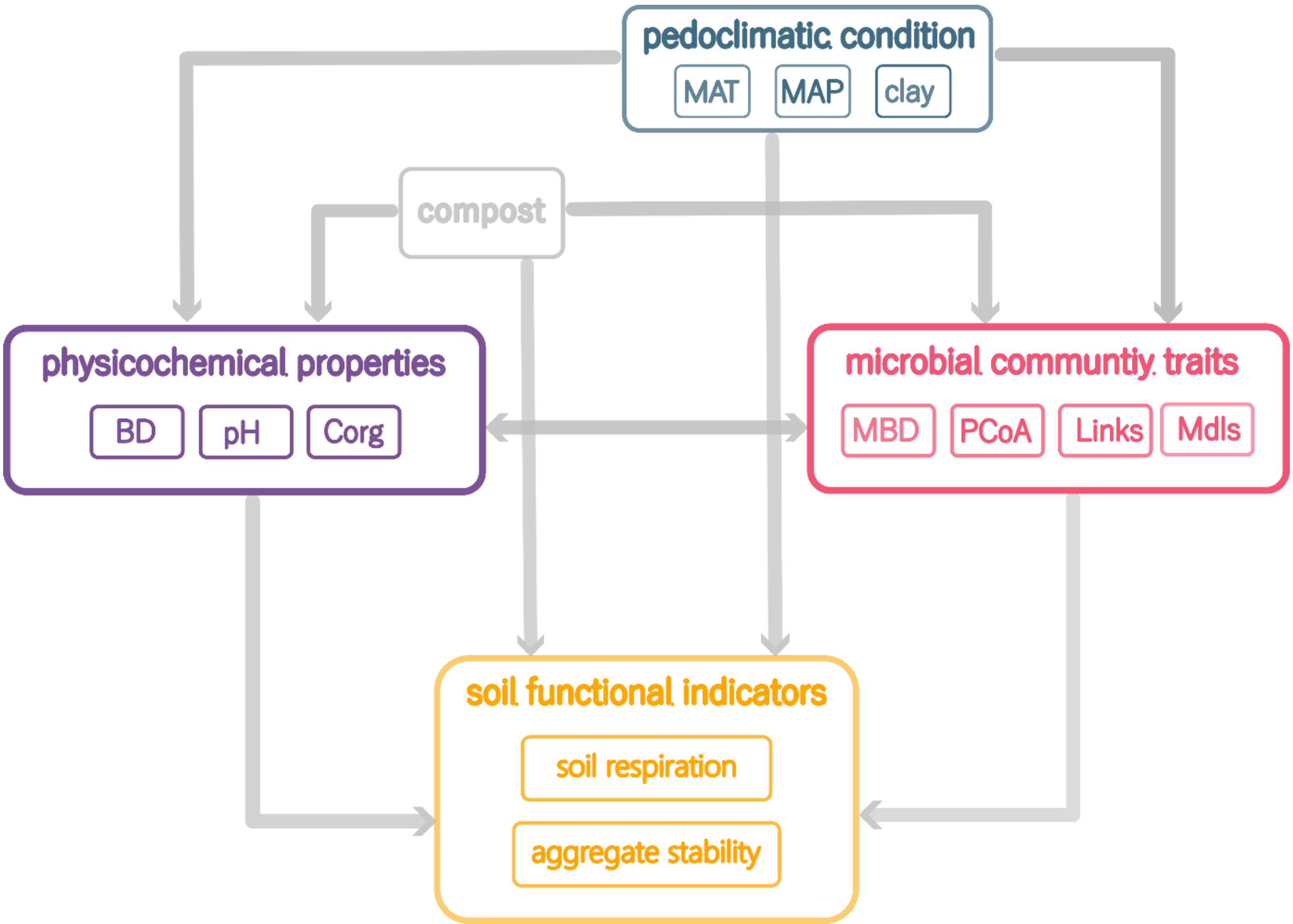

Figure S2 A priori structural equation model illustrating the direct and indirect effects of pedoclimatic conditions (mean annual temperature (MAT), mean annual precipitation (MAP), and clay content), compost application, manageable physicochemical soil properties (SOC, pH, bulk density), and microbial community traits (diversity (MDB), composition (PCoA), association complexity (links), and the relative abundance of four modules) on soil functional proxies (aggregate stability and soil respiration).

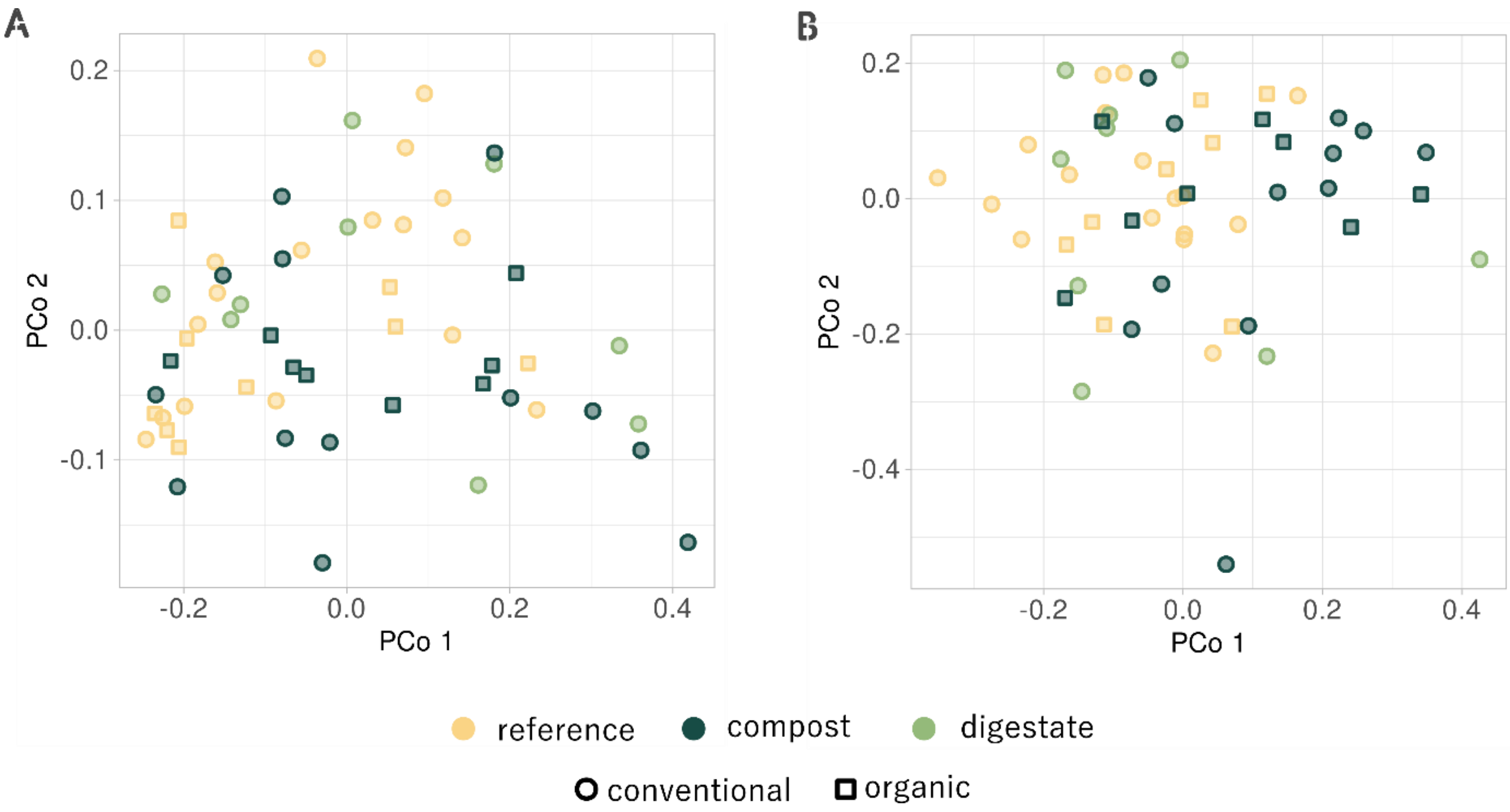

Figure S3 Bacterial and fungal community structure as affected by management system and fertiliser regimes. Ordination plots display bacterial (A) and fungal (B) community structures using Principal Coordinates Analysis (PCoA). Different symbols represent management systems, while colours denote fertiliser regimes.

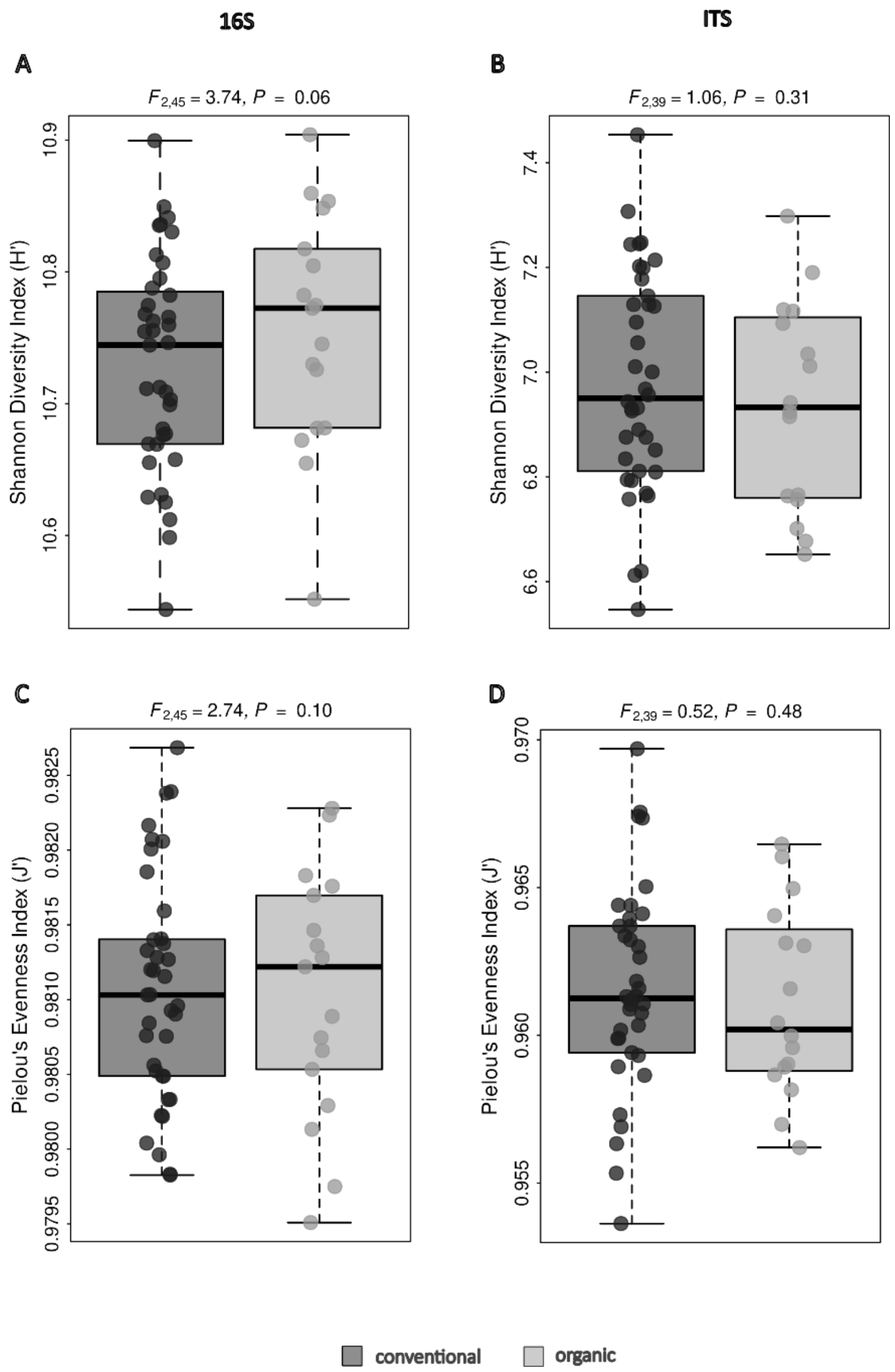

Figure S4 Bacterial and fungal diversity as influenced by a management system. Box plots illustrate the impact of conventional and organic management systems on diversity, as measured by the Shannon-Wiener Diversity Index, and evenness, as measured by Pielou's Evenness Index for bacterial (A, C) and fungal (B, D) communities. The F-statistic and corresponding p-value from the linear model are presented above each box, indicating the statistical significance of the effect of the management system on diversity and evenness.

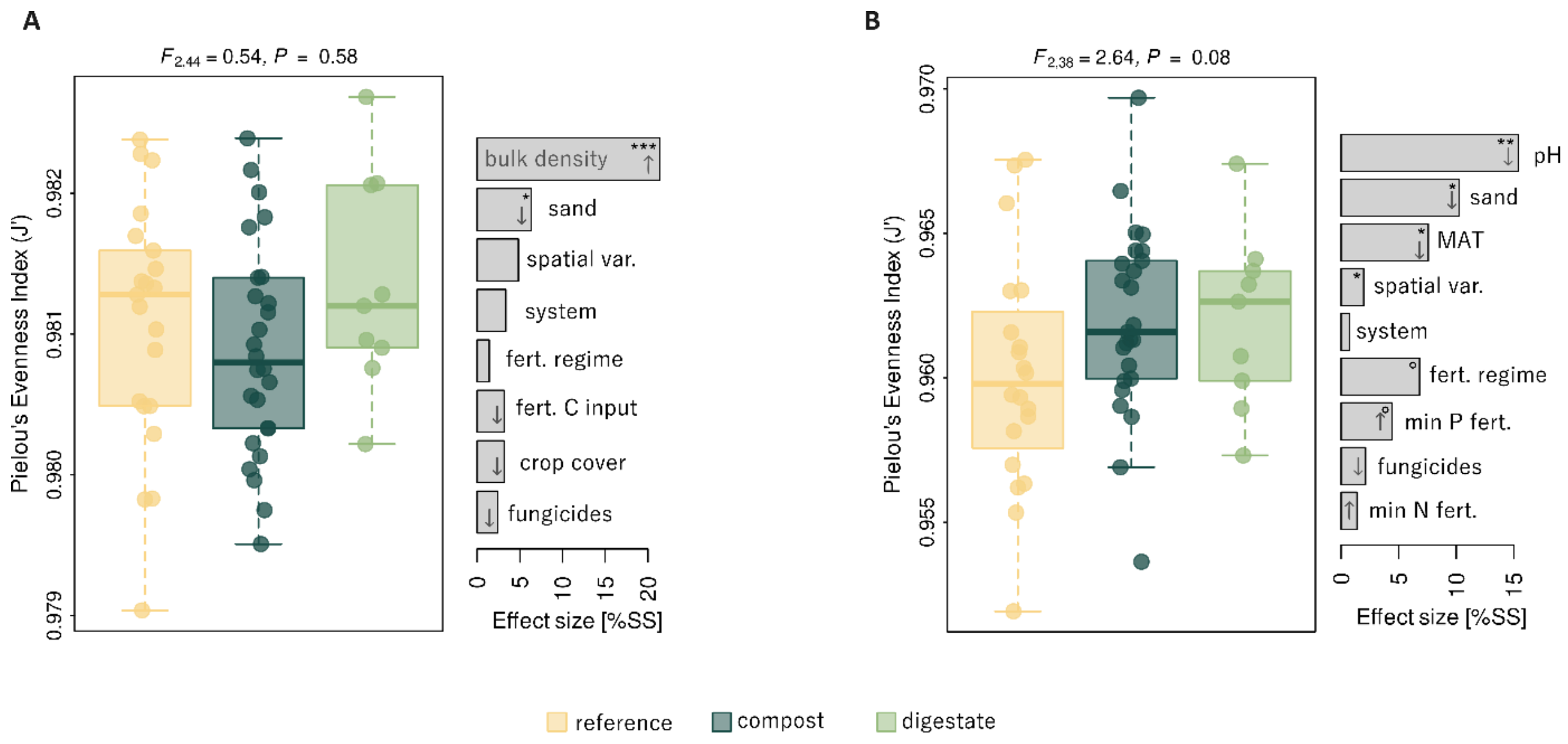

Figure S5 Bacterial and fungal evenness as influenced by management and pedoclimatic conditions. Box plots show the impact of different fertiliser regimes (reference, compost, and digestate) on soil bacterial (A) and fungal (B) evenness, measured using Pilou's Evenness Index. The F-statistic and corresponding p-value from the linear model is presented above each box, indicating the statistical significance of the effect of the fertiliser regime on diversity. Different letters above the boxplots denote significant differences between fertiliser regimes. Horizontal bar plots illustrate the primary pedoclimatic and management drivers of bacterial and fungal diversity, respectively, as percentages of the total sum of squares (SS). The direction of the effect, whether positive or negative, is indicated with arrows. Significance levels are indicated by stars (* $p < 0.05$, ** $p < 0.01$, *** $p < 0.001$).

A

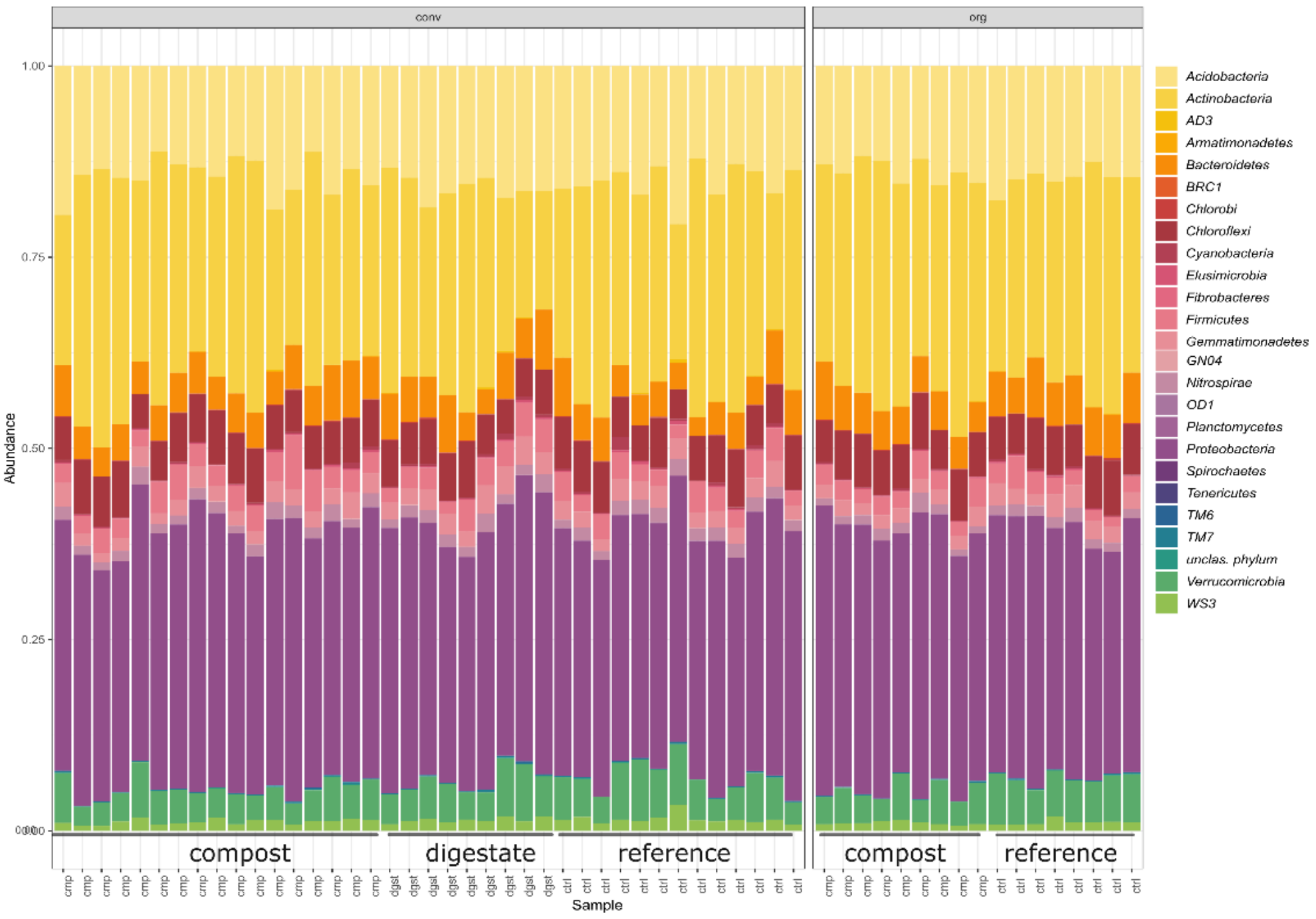

B

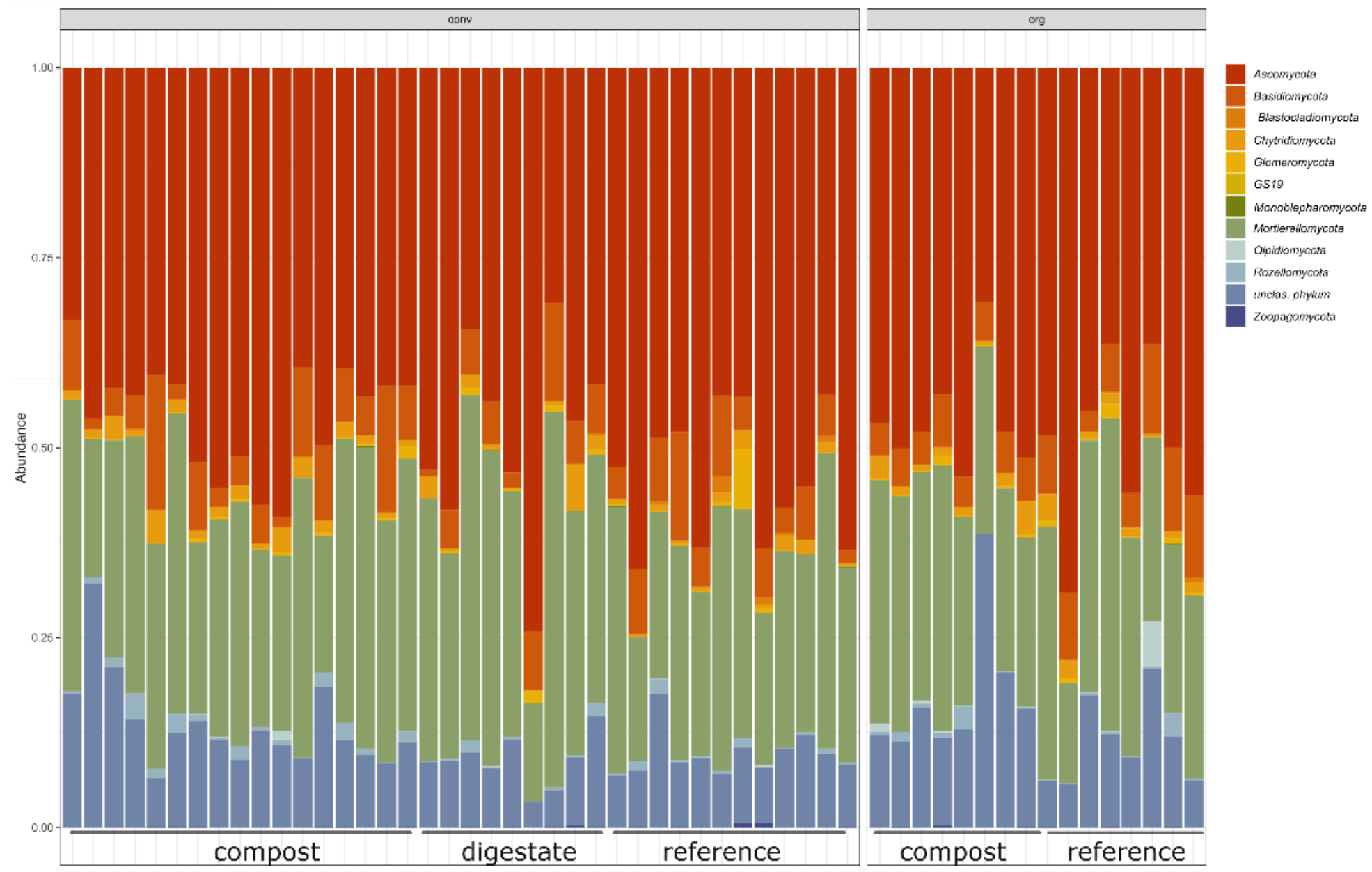

Fig. S6 Bacterial and fungal taxonomic composition in soils under various management and fertiliser regimes. Panels (A) and (B) illustrate the taxonomic composition of the soil bacterial and fungal communities, respectively, in terms of the relative abundance of different phyla. "Conv" denotes fields under conventional management, and "Org" denotes fields under organic management. Sample names indicate the corresponding fertiliser regime: "ctrl" (reference), "cmp" (compost), and "dgst" (digestate).

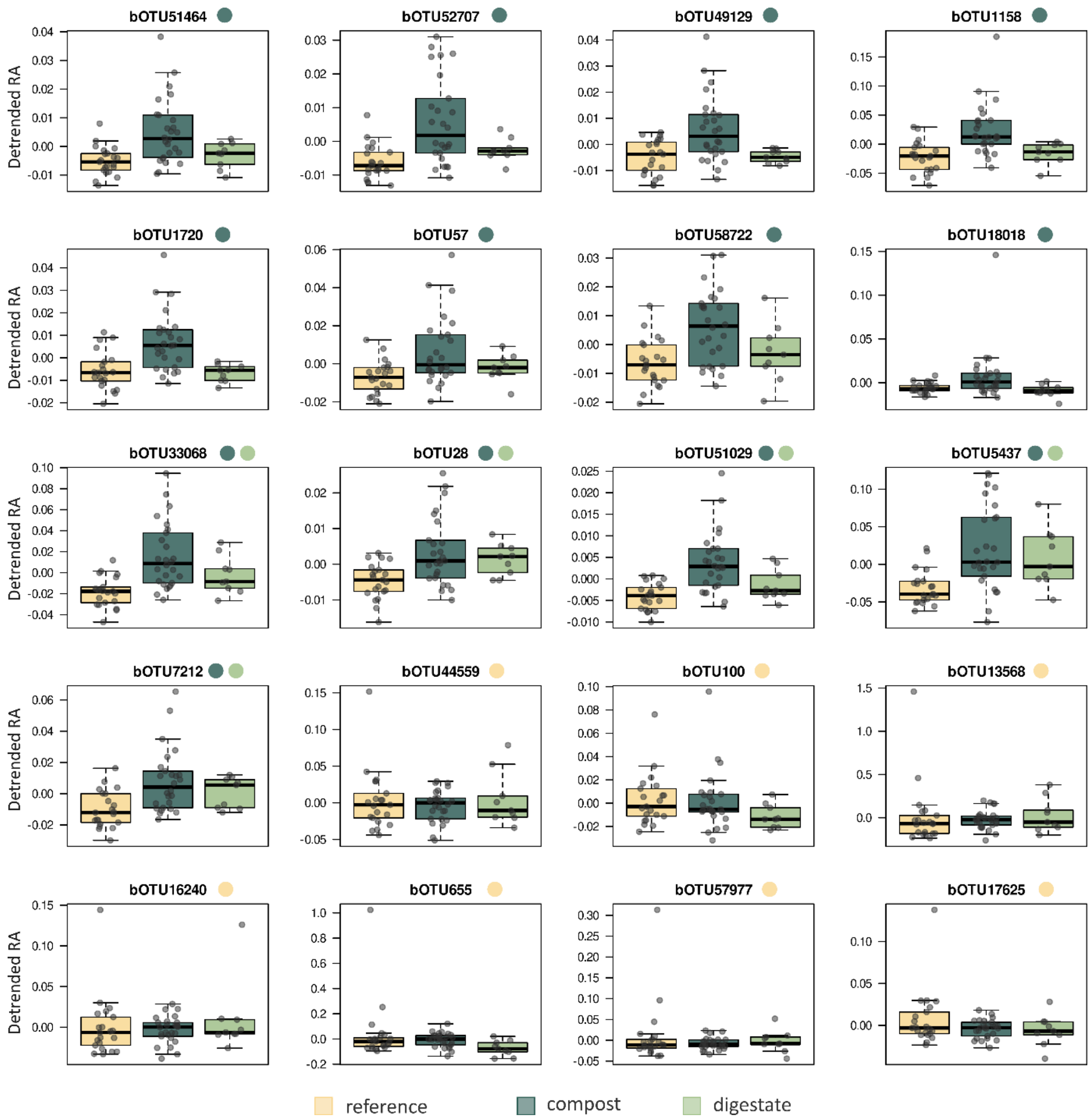

Figure S7 Fertiliser-sensitive bacteria taxa. Box plots show 16S-OTUs that were differentially abundant across fertiliser regimes. The y-axis shows the relative abundance (RA) of each OTU, detrended for correction variables: mean annual precipitation, bulk density, soil texture, and pH. Colours represent the abundance under different fertiliser regimes. Coloured dots beside the OTU name indicate which fertiliser regime the taxa were significantly more abundant ($p<0.05$, log2fold change > 2).

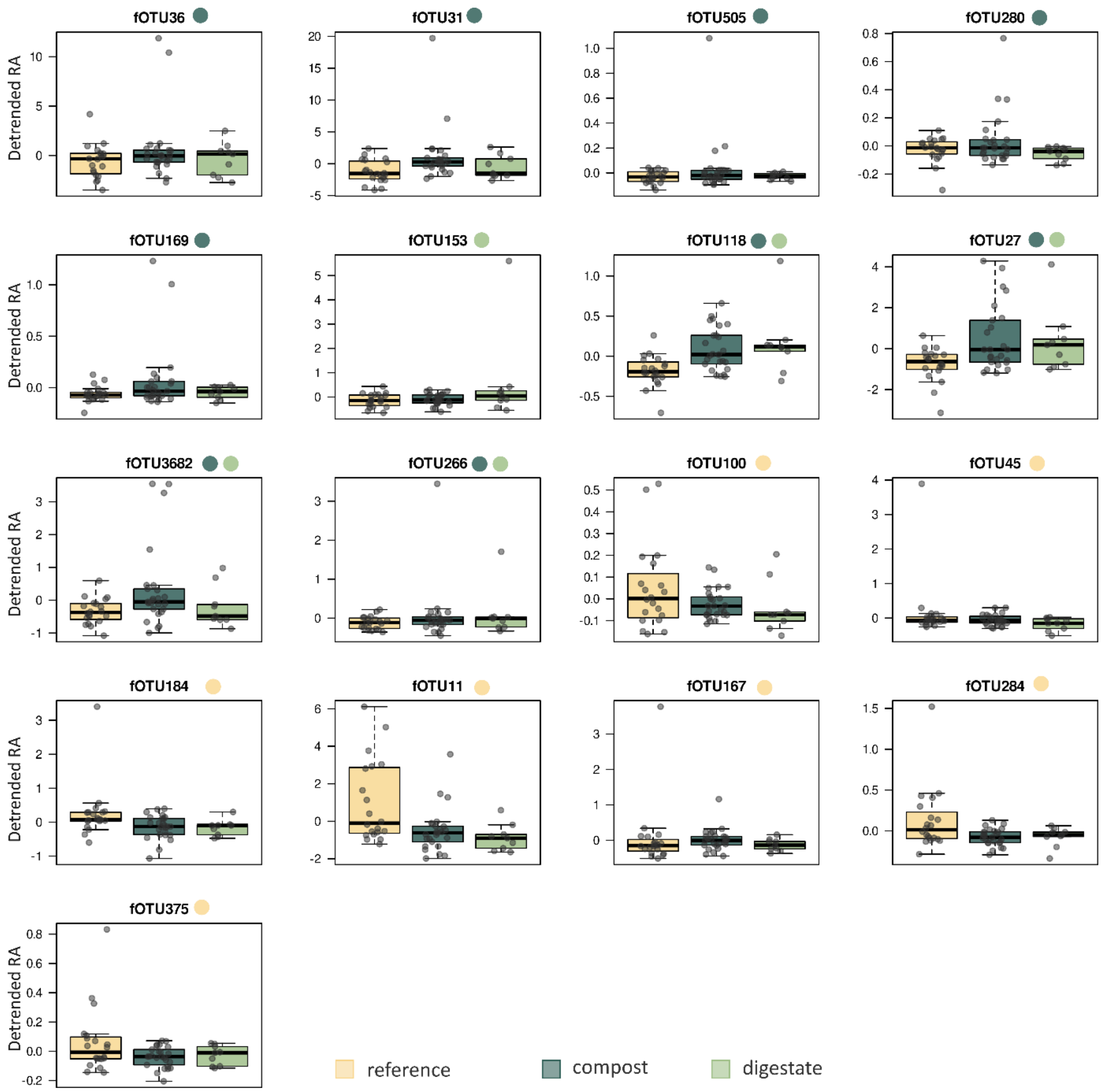

Figure S8 Fertiliser-sensitive microbial fungi. Box plots show ITS-OTUs that were differentially abundant across fertiliser regimes. The y-axis indicates the relative abundance (RA) of each OTU, detrended for correction variables: mean annual precipitation, bulk density, soil texture, and pH. Colours represent the abundance under different fertiliser regimes. Coloured dots beside the OTU name indicate which fertiliser regime the taxa were more significantly abundant ($p<0.05$, log2fold change > 2).

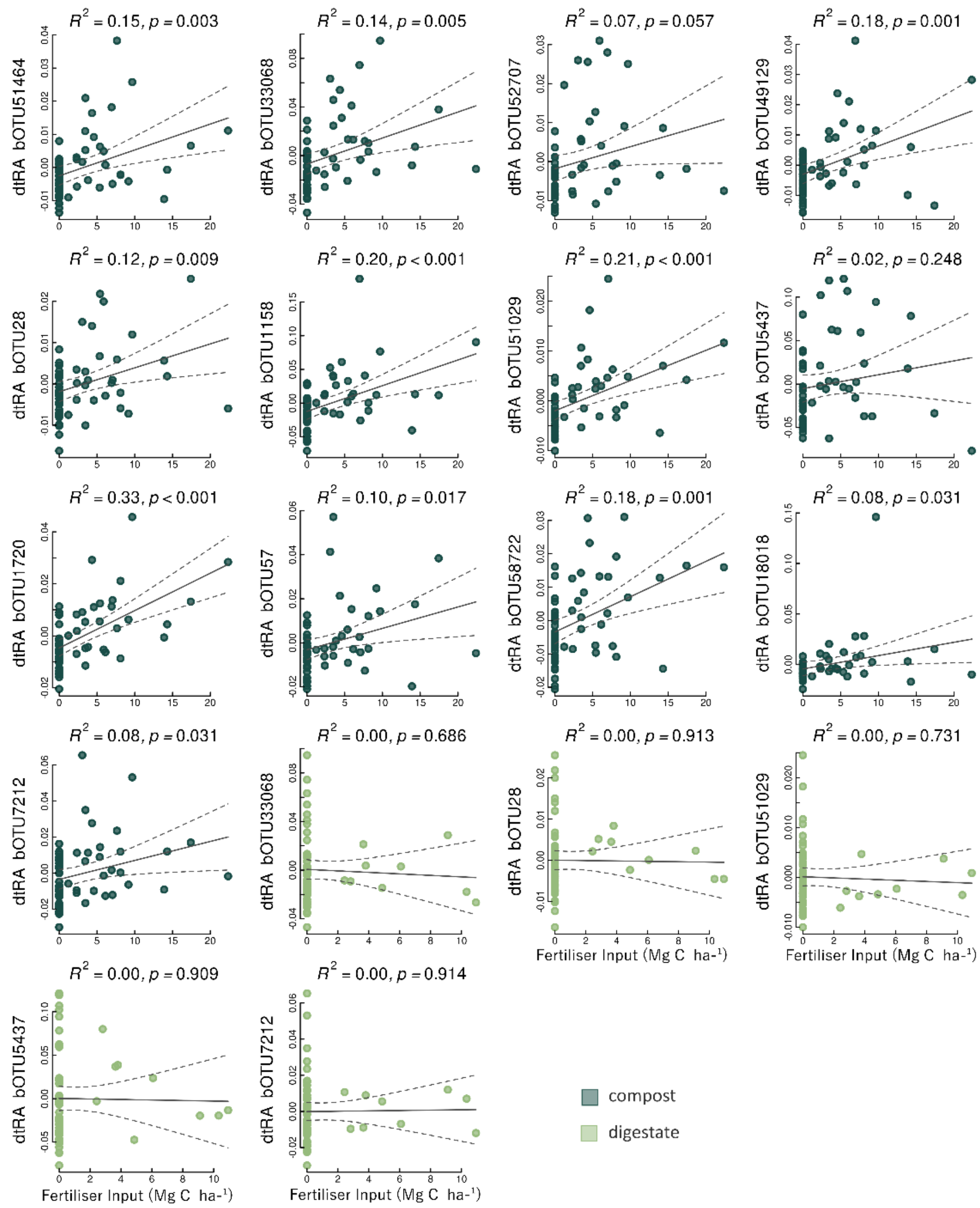

Figure S9 Compost- and digestate-sensitive bacterial OTUs. Scatterplots illustrate the linear relationship between the detrended relative abundance (dtRA) of differentially abundant OTUs and the cumulative amount of compost or digestate applied during the five years preceding sampling. Relative abundances were detrended for mean annual precipitation, bulk density, soil texture, and pH. Points are colour-coded by fertiliser regime, and model performance metrics (adjusted R^2 and P-value) are shown at the top of each panel.

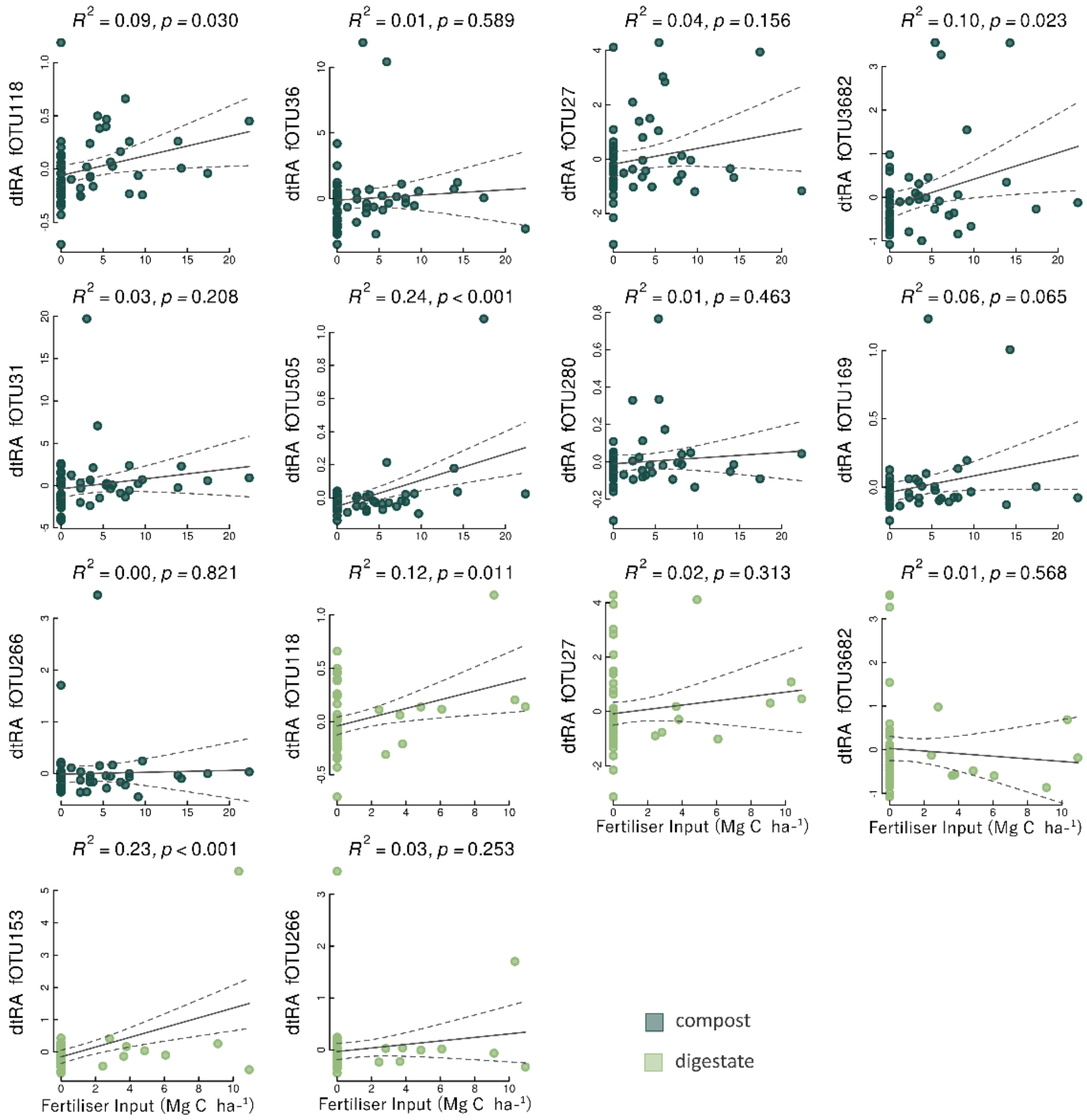

Figure S10 Compost- and digestate-sensitive fungal OTUs. Scatterplots illustrate the linear relationship between the detrended relative abundance (dtRA) of differentially abundant OTUs and the cumulative amount of compost or digestate applied during the five years preceding sampling. Relative abundances were detrended for mean annual precipitation, bulk density, soil texture, and pH. Points are colour-coded by fertiliser regime, and model performance metrics (adjusted R^2 and P-value) are shown at the top of each panel.

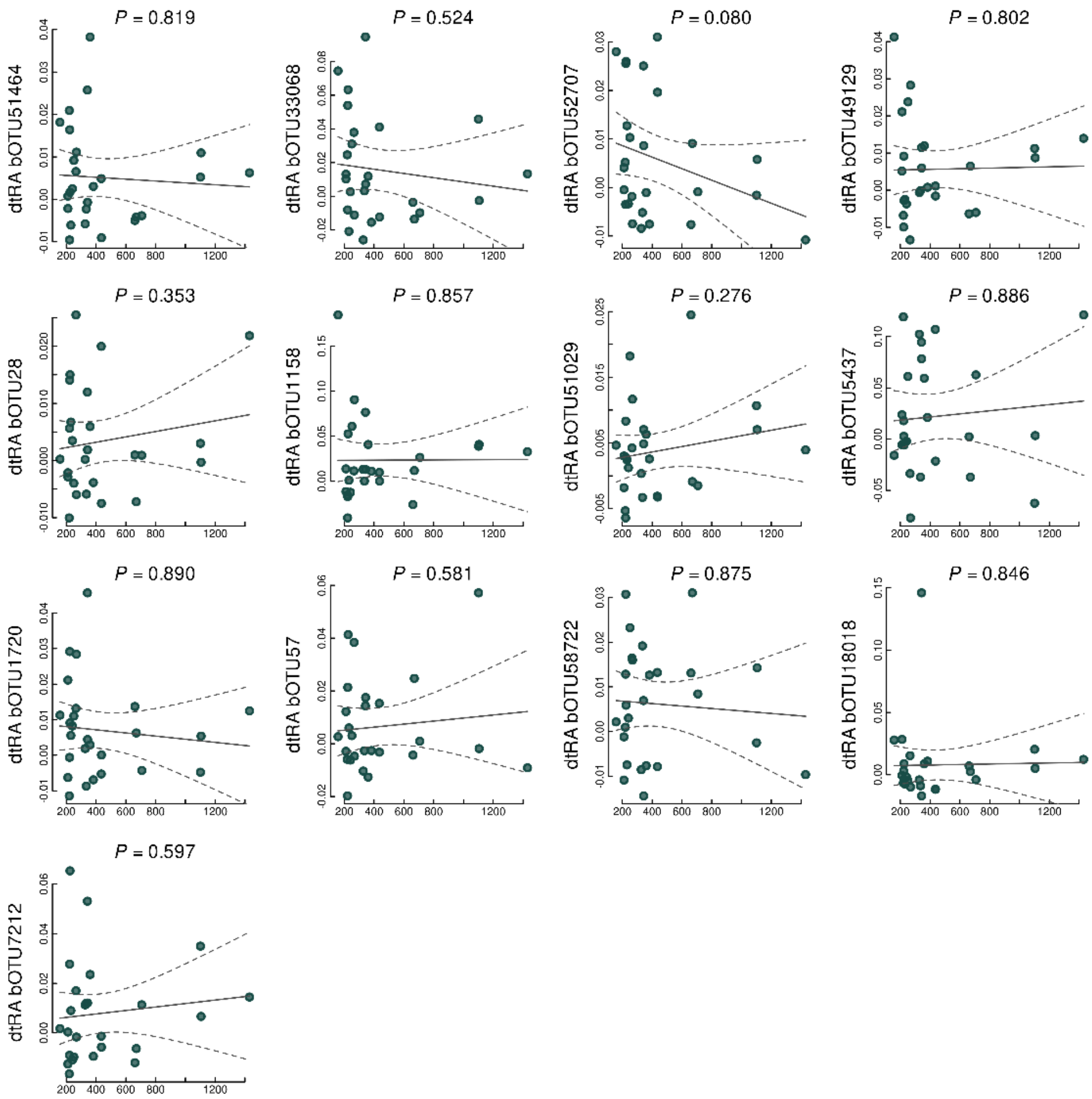

Fig. S11 Compost-sensitive bacterial OTUs and time since last application. Scatterplots illustrate the linear relationship between the detrended relative abundance (dtRA) of compost-sensitive bacterial OTUs and the interval between the last fertilisation event and soil sampling, within compost-amended fields. Relative abundances were detrended for mean annual precipitation, bulk density, soil texture, and pH. Model performance metrics (uncorrected P-value) are shown at the top of each panel; models included the cumulative quantity of compost applied as a covariate.

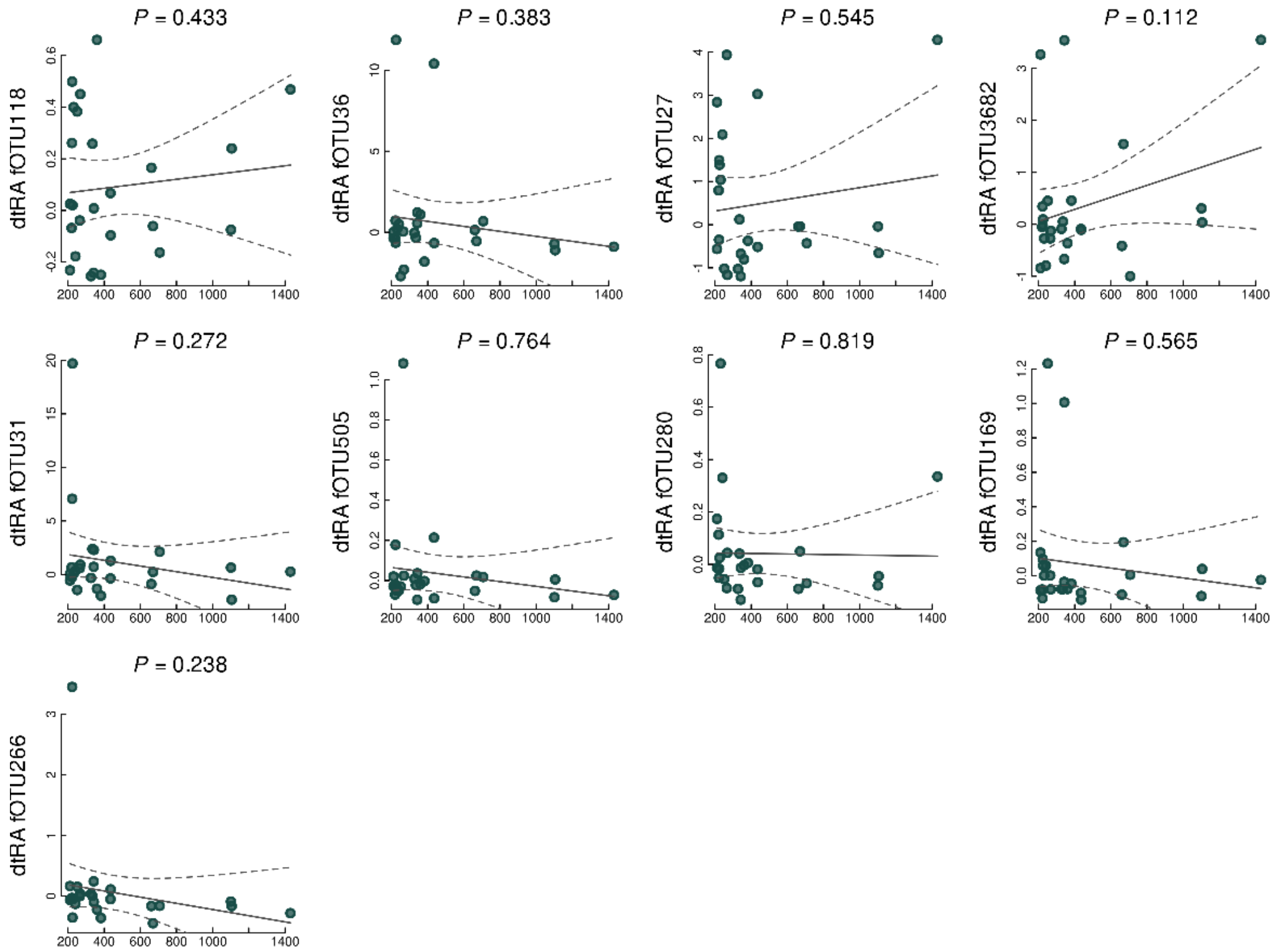

Fig. S12 Compost-sensitive fungal OTUs and time since last application. Scatterplots illustrate the linear relationship between the detrended relative abundance (dtRA) of compost-sensitive fungal OTUs and the interval between the last fertilisation event and soil sampling, within compost-amended fields. Relative abundances were detrended for mean annual precipitation, bulk density, soil texture, and pH. Model performance metrics (uncorrected *P*-value) are shown at the top of each panel; models included the cumulative quantity of compost applied as a covariate.

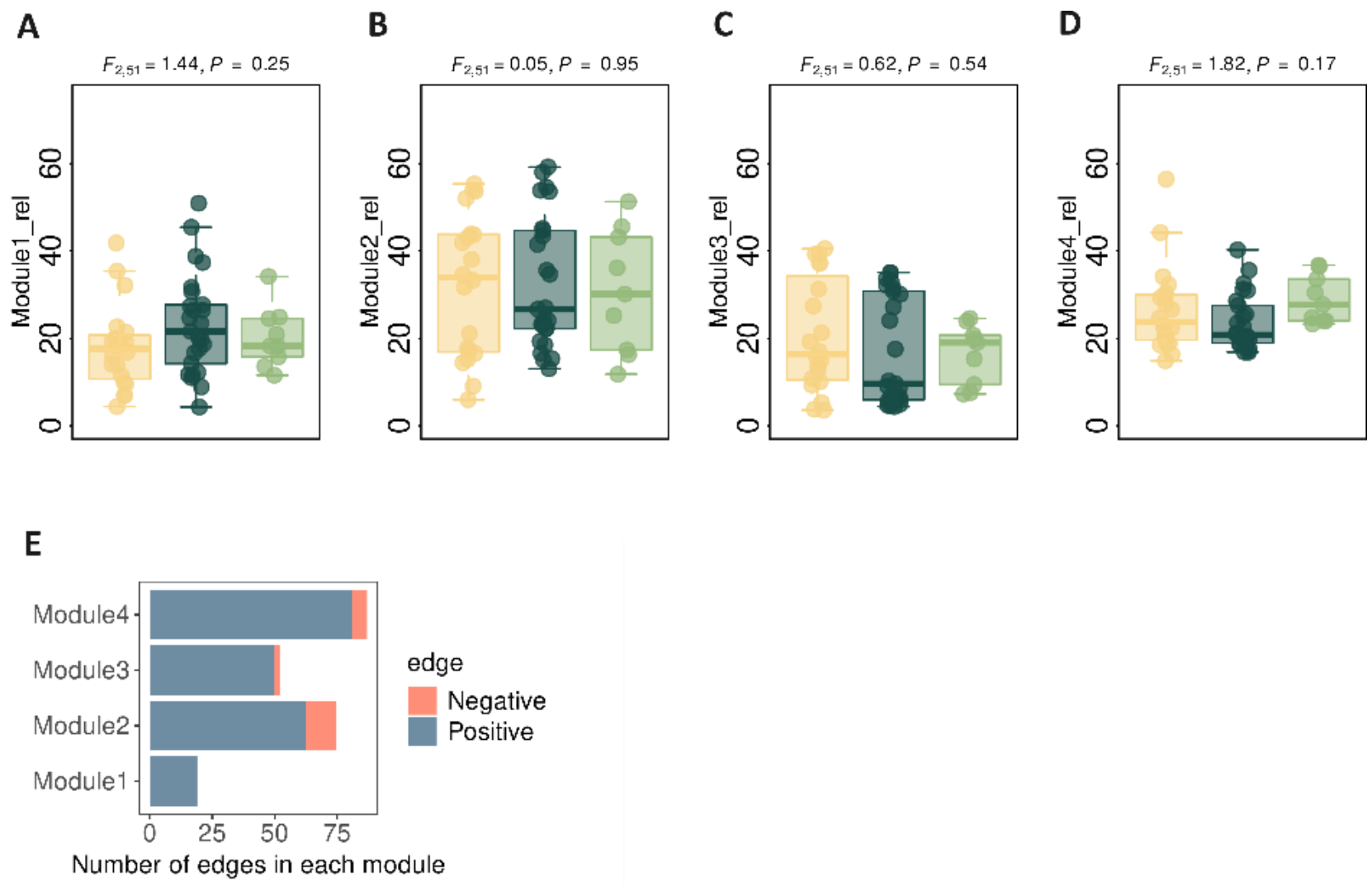

Fig. S13 Relative abundance and composition of network modules. (A-D) Box plots depict the cumulative relative abundance of taxa in modules 1–4 across different fertiliser regimes. The F-statistic and p-value from the linear model testing the fertiliser effect are shown above each plot. Different letters mark significant pairwise differences between regimes (Tukey HSD, $p < 0.05$). . (E) Number of positive and negative edges per module.

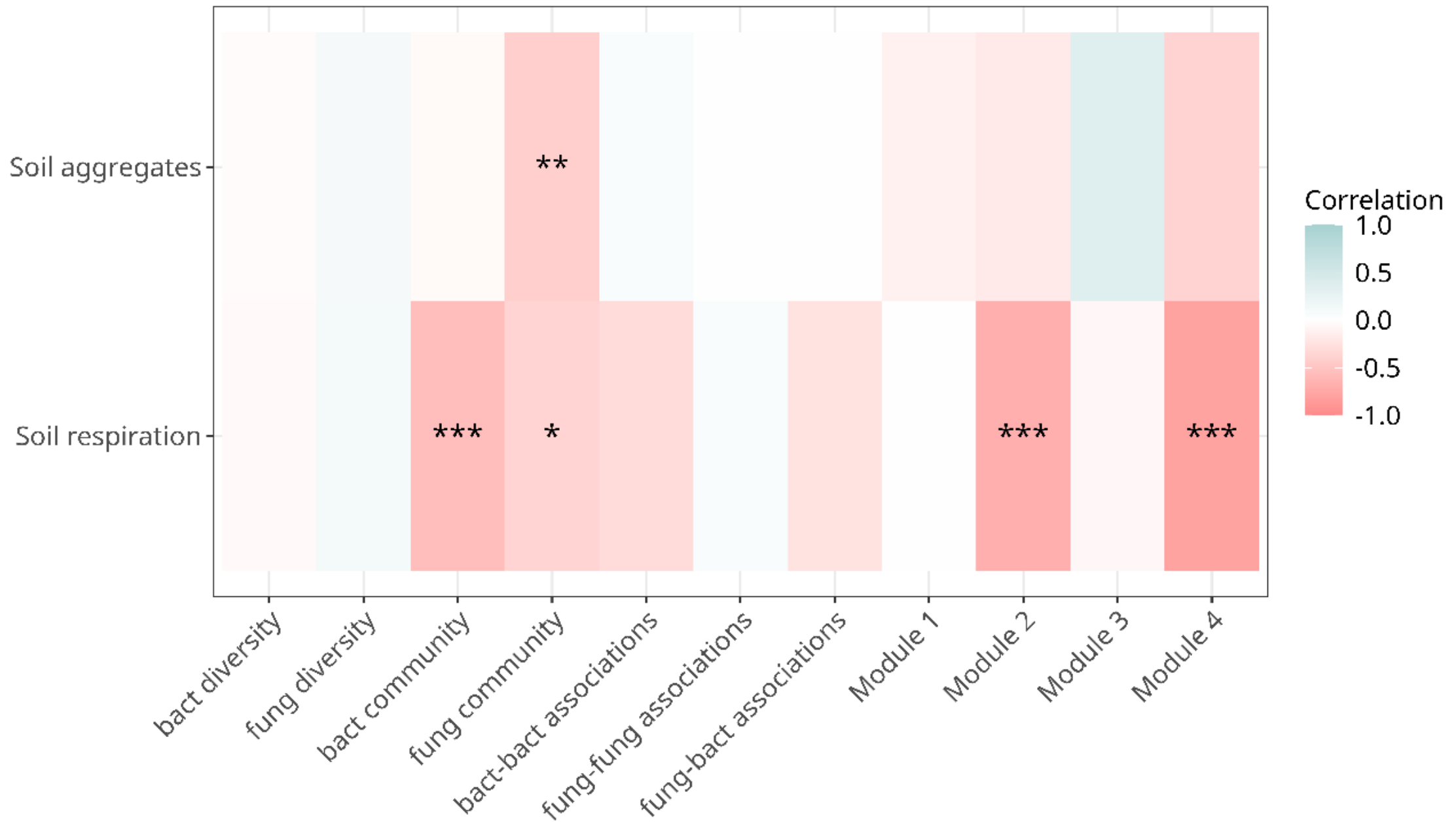

Fig. S14 Correlations between microbial community traits and soil functions. Heat-map of Spearman's ρ coefficients, coloured red for negative and blue for positive associations between microbial community traits and soil-function proxies. P-values from two-sided tests were adjusted for multiple comparisons with the false discovery rate. Significance levels: * $p < 0.05$, ** $p < 0.01$, *** $p < 0.001$.

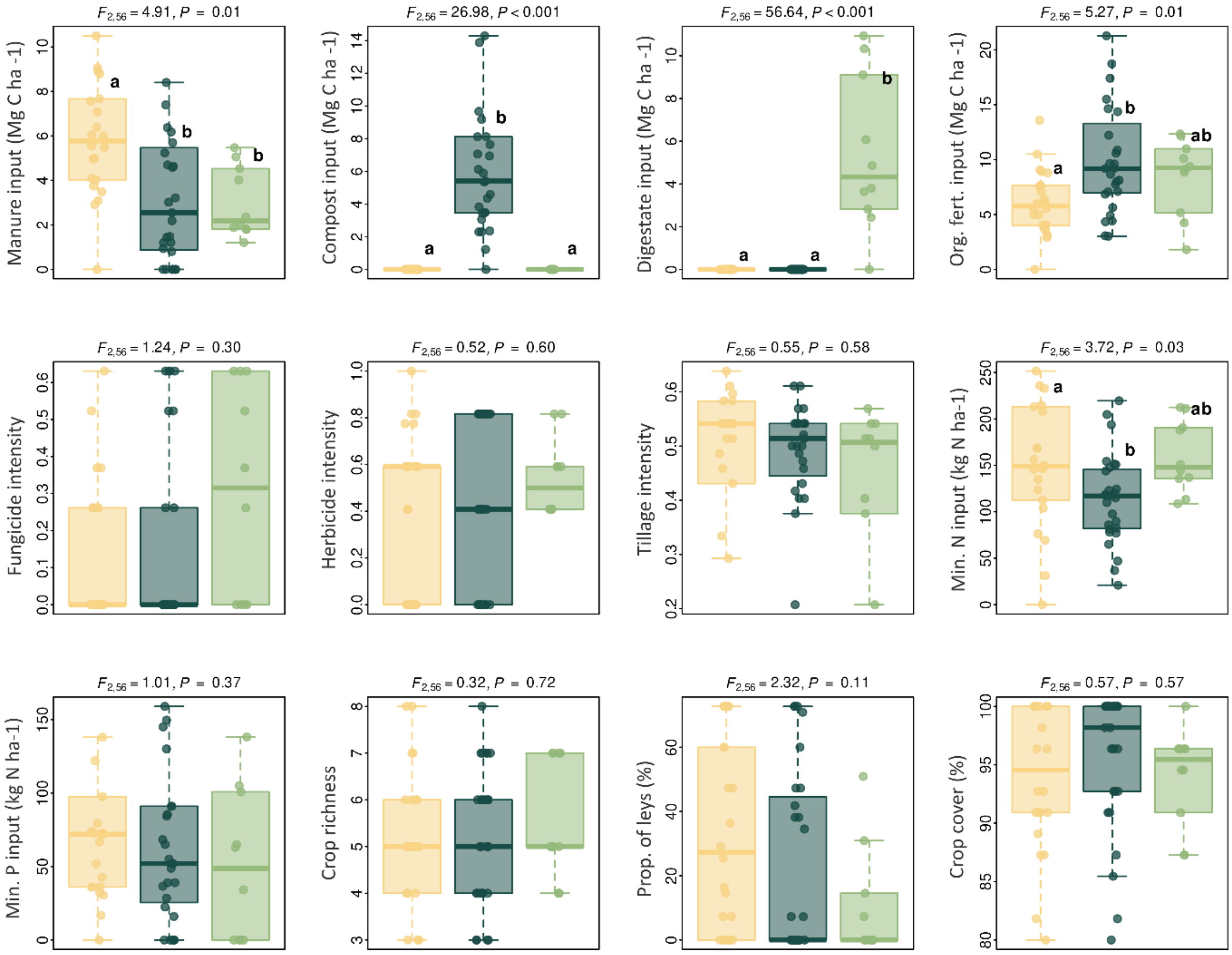

Fig. S15 Management variables across fertiliser regimes. All variables were assessed over the five years preceding sampling, except for fungicide and herbicide intensity, which were calculated for the year immediately prior. The F-statistic and p-value from the linear model testing the fertiliser effect are shown above each plot. Different letters indicate significant differences between regimes (Tukey HSD, $p < 0.05$).

Supplementary Tables

Table S1 P-values and per cent sum of squares (% SS) for factors in the PERMANOVA of bacterial (16S) and fungal (ITS) communities.

	16S		ITS	
	p-value	% SS	p-value	% SS
**spatial var.**	**0.002**	**3.62**	**0.008**	**7.31**
MAP	0.383	0.9	0.019	1.65
**MAT**	**0.096**	**1.53**	0.238	1.11
altitude	0.474	0.79	0.143	1.23
**bd**	**0.001**	**4.27**	**0.001**	**2.76**
**clay**	**0.001**	**5.06**	**0.002**	**2.16**
**sand**	**0.004**	**3.19**	**0.001**	**2.39**
**ph**	**0.001**	**11.67**	**0.001**	**3.5**
**Mang. system**	0.132	0.88	**0.006**	**1.55**
**Fert. regime**	0.097	1.81	**0.002**	**2.85**
fert_Ci	0.259	0.66	0.837	0.55
fym_Ci	0.284	0.66	0.158	1
comp_Ci	0.146	0.86	0.596	0.68
dig_Ci	0.26	0.68	0.404	0.76
fung_int	0.422	0.54	0.466	0.75
**herb_int**	0.193	0.79	**0.009**	**1.54**
till_int	0.271	0.65	0.979	0.42
fert_minN	0.4	0.58	0.558	0.7
fert_minP	0.705	0.43	0.926	0.49
crop div	0.275	0.67	0.443	0.76
leys	0.163	0.84	0.171	0.94
**crop cover**	**0.069**	**1.13**	0.275	0.88

Environmental variables (spatial var., MAP, MAT, altitude, bd, clay, sand, pH) were entered first; all other factors were fitted sequentially after adjustment for these covariates. Percentage of total sum of squares from sequential PERMANOVA indicating each predictor's unique contribution to community composition variation. Effect direction is indicated by standardised coefficients. spatial var., spatial variables (PCNM eigenvectors); MAP, mean annual precipitation; MAT, mean annual temperature; bd, bulk density; clay, sand, percent soil texture; pH, soil pH in water; System, management system (conventional vs. organic); Treatment, fertiliser regime (reference, compost, digestate); fert_Ci, total organic-C input; fym_Ci, organic-C input from farmyard manure; comp_Ci, organic-C input from compost; dig_Ci, organic-C input from solid digestate; fung_int, herb_int, till_int, intensity indices for fungicide, herbicide, and tillage (scaled events × depth or product count); fert_minN, fert_minP, mineral N and P inputs; crop div, crop-rotation richness; leys, proportion of leys; crop cover, proportion of soil cover.

Table S2 Parameter estimates, p-values, and percent sum of squares (% SS) for factors in the multivariate model of bacterial diversity (MBD) and evenness (MEV).

	MBD			MEV		
	Estimate	p-value	%SS	Estimate	p-value	%SS
spatial var.	-0.01	0.570	0.61	<0.01	0.105	4.84
MAP	0.01	0.173	3.3	<0.01	0.349	1.66
MAT	-0.07	0.101	4.81	<0.01	0.883	0.04
altitude	<0.01	0.162	3.47	<0.01	0.196	3.18
bd	0.07	0.138	4.03	**0.002**	**<0.001**	**21.4**
clay	-0.01	0.430	1.12	<0.01	0.207	2.2
sand	<0.01	0.090	5.31	**<0.01**	**0.035**	**6.32**
ph	0.01	0.661	0.35	<0.01	0.227	2.01
Mang. system		**0.060**	**5.66**		0.105	3.38
Fert. regime		0.992	0.03		0.584	1.43
fert_Ci	0.01	0.209	2.57	<0.01	0.117	3.17
fym_Ci	-0.01	0.134	3.63	<0.01	0.288	1.48
comp_Ci	<0.01	0.648	0.35	<0.01	0.213	2.02
dig_Ci	<0.01	0.776	0.13	<0.01	0.534	0.51
fung_int	-0.04	0.479	0.83	<0.01	0.172	2.42
herb_int	**-0.09**	**0.027**	**7.65**	<0.01	0.186	2.28
till_int	-0.02	0.782	0.13	<0.01	0.994	0
fert_minN	<0.01	0.152	3.33	<0.01	0.328	1.25
fert_minP	<0.01	0.300	1.76	<0.01	0.357	1.12
crop div	0.01	0.407	1.13	<0.01	0.837	0.06
leys	<0.01	0.850	0.06	<0.01	0.354	1.13
crop cover	**0.01**	**0.026**	**7.73**	<0.01	0.117	3.17

Environmental variables (spatial var., MAP, MAT, altitude, bd, clay, sand, pH) were entered first; all other factors were fitted sequentially after adjustment for these covariates. Percentage of total sum of squares from sequential ANOVA indicating each predictor's unique contribution to community composition variation. Effect direction is indicated by standardised coefficients. MBD, mean bacterial diversity (Shannon index); MEV, Pielou's evenness; spatial var., spatial variables (PCNM eigenvectors); MAP, mean annual precipitation; MAT, mean annual temperature; bd, bulk density; clay, sand, percent soil texture; pH, soil pH in water; System, management system (conventional vs. organic); Treatment, fertiliser regime (reference, compost, digestate); fert_Ci, total organic-C input; fym_Ci, organic-C input from farmyard manure; comp_Ci, organic-C input from compost; dig_Ci, organic-C input from solid digestate; fung_int, herb_int, till_int, intensity indices for fungicide, herbicide, and tillage (scaled events × depth or product count); fert_minN, fert_minP, mineral N and P inputs; crop div, crop-rotation richness; leys, proportion of leys; crop cover, proportion of soil cover.

Table S3 Parameter estimates, p-values, and percent sum of squares (% SS) for factors in the multivariate model of fungal diversity (MBD) and evenness (MEV).

	MBD			MEV		
	Estimate	p-value	%SS	Estimate	p-value	%SS
spatial var.	-0.003	0.34	0.001	<0.001	0.038	1.96
MAP	<0.001	0.20	2.99	<0.001	0.046	7.57
MAT	-0.013	0.84	0.07	0.002	0.575	0.57
altitude	<0.001	0.72	0.23	<0.001	0.805	0.11
bd	0.074	0.42	1.09	-0.002	0.659	0.29
clay	-0.008	0.71	0.22	>-0.001	0.785	0.11
sand	-0.008	0.04	7.16	>-0.001	0.011	10.26
ph	-0.090	0.14	3.69	-0.008	0.002	15.37
Mang. system	NA	0.31	1.77	NA	0.476	0.73
Fert. regime	NA	0.01	15.62	NA	0.084	6.81
fert_Ci	<0.001	0.98	0	<0.001	0.561	0.49
fym_Ci	-0.006	0.60	0.47	>-0.001	0.670	0.26
comp_Ci	0.004	0.51	0.76	>-0.001	0.696	0.22
dig_Ci	-0.009	0.48	0.85	>-0.001	0.396	1.04
fung_int	-0.031	0.81	0.1	-0.006	0.221	2.13
herb_int	0.057	0.55	0.61	0.002	0.642	0.31
till_int	0.070	0.69	0.28	-0.002	0.814	0.08
fert_minN	<0.001	0.66	0.33	<0.001	0.327	1.38
fert_minP	<0.001	0.49	0.81	<0.001	0.075	4.42
crop div	-0.010	0.65	0.36	>-0.001	0.697	0.22
leys	<0.001	0.75	0.18	<0.001	0.990	0
crop cover	0.005	0.33	1.61	<0.001	0.467	0.76

Environmental variables (spatial var., MAP, MAT, altitude, bd, clay, sand, pH) were entered first; all other factors were fitted sequentially after adjustment for these covariates. Percentage of total sum of squares from sequential ANOVA indicating each predictor's unique contribution to community composition variation. Effect direction is indicated by standardised coefficients. MBD, mean bacterial diversity (Shannon index); MEV, Pielou's evenness; spatial var., spatial variables (PCNM axes); MAP, mean annual precipitation; MAT, mean annual temperature; bd, bulk density; clay, sand, percent soil texture; pH, soil pH in water; System, management system (conventional vs. organic); Treatment, fertiliser regime (reference, compost, digestate); fert_Ci, total organic-C input; fym_Ci, organic-C input from farmyard manure; comp_Ci, organic-C input from compost; dig_Ci, organic-C input from solid digestate; fung_int, herb_int, till_int, intensity indices for fungicide, herbicide, and tillage (scaled events × depth or product count); fert_minN, fert_minP, mineral N and P inputs; crop div, crop-rotation richness; leys, proportion of leys; crop cover, proportion of soil cover.

Table S4 Differentially abundant bacterial taxa across fertiliser regimes identified by DESeq2.

	phylum	*class*	*order*	*family*	*genus*	*species*	*fert. regime*	*module*	*prevalence*
bOTU51464	Actinobacteria	Actinobacteria	Actinomycetales	Streptosporangiaceae			cmp		48.2
bOTU52707	Actinobacteria	Actinobacteria	Actinomycetales				cmp		58.9
bOTU49129	Actinobacteria	Actinobacteria	Actinomycetales				cmp		51.8
bOTU1158	Proteobacteria	Alphaproteobacteria	Rhizobiales	Hyphomicrobiaceae			cmp	module1	85.7
bOTU1720	Actinobacteria	Actinobacteria	Actinomycetales	Thermomonosporaceae	Actinomadura	A. vinacea	ctrl		51.8
bOTU57	Firmicutes	Bacilli	Bacillales	Thermoactinomycetaceae	Planifilum		cmp		73.2
bOTU58722	Bacteroidetes	[Saprospirae]	[Saprospirales]	Chitinophagaceae			cmp		69.6
bOTU18018	Firmicutes	Bacilli	Bacillales	Paenibacillaceae			cmp		53.6
bOTU33068	Firmicutes	Bacilli	Bacillales	Bacillaceae	Geobacillus		cmp/dgst	module1	76.8
bOTU28	Firmicutes	Clostridia	Clostridiales	Clostridiaceae	Clostridium		cmp/dgst		44.6
bOTU51029	Chloroflexi	Thermomicrobia	Sphaerobacterales				cmp/dgst		42.9
bOTU5437	Proteobacteria	Gammaproteobacteria	Pseudomonadales	Pseudomonadaceae	Pseudomonas		cmp/dgst		98.2
bOTU7212	Firmicutes	Bacilli	Bacillales	Planococcaceae	Ureibacillus		cmp/dgst		83.9
bOTU44559	Proteobacteria	Alphaproteobacteria	Ellin329				ref	module4	35.7
bOTU100	Proteobacteria	Gammaproteobacteria	Xanthomonadales	Sinobacteraceae			ref		41.1
bOTU13568	Acidobacteria	Acidobacteriia	Acidobacteriales	Koribacteraceae			ref	module4	75.0
bOTU16240	Acidobacteria	Acidobacteriia	Acidobacteriales	Acidobacteriaceae	Edaphobacter	E.modestum	ref	module4	41.1
bOTU655	Cyanobacteria	Oscillatoriophycideae	Oscillatoriales	Phormidiaceae	Phormidium		ref		46.4
bOTU57977	Bacteroidetes	Cytophagia	Cytophagales	Cytophagaceae			ref		37.5
bOTU17625	Acidobacteria	Sva0725	Sva0725				ref		44.6

Only taxa with significant changes in relative abundance (adjusted $p < 0.05$) are shown. 'ref', 'cmp' and 'dgst' denote reference, compost and digestate fertiliser regimes, respectively. Module assignment refers to co-occurrence network modules. Prevalence denotes the proportion of the 56 surveyed farms at which the OTU was detected.

Table S5 Bacterial taxa enriched by fertilizer regimes.

fertiliser regime	*taxa*	*observed*	*expected*	*pValue*	*FDR*
compost	Firmicutes	5	0.374322493	2.14E-05	0.000513086
compost	Bacilli	4	0.21944632	5.76E-05	0.004781338
compost	Bacillales	4	0.236722307	7.73E-05	0.010286619
digestate	Firmicutes	3	0.14397	0.000224	0.005386

Table S6 Differentially abundant fungal taxa across fertiliser regimes identified by DESeq2.

	phylum	*class*	*order*	*family*	*genus*	*species*	*fert. regime*	*module*	*prevalence*	*guild*
fOTU36	Ascomycota	Pezizomycetes	Pezizales	Pyronemataceae			cmp	module 1	66.7	Sap-Symb
fOTU31							cmp		48.1	
fOTU505	Ascomycota						cmp		25.9	
fOTU280							cmp		29.6	
fOTU169	Basidiomycota	Tremellomycetes	Cystofilobasidiales	Mrakiaceae	Mrakia	M. frigida	cmp		53.7	Sap
fOTU153	Ascomycota	Leotiomycetes	Erysiphales	Erysiphaceae	Blumeria		dgst		48.1	Path
fOTU118	Ascomycota	Sordariomycetes	Microascales				cmp/dgst	module 1	90.7	
fOTU27	Ascomycota						cmp/dgst	module 1	92.6	
fOTU3682	Basidiomycota	Tremellomycetes					cmp/dgst	module 2	63.0	
fOTU266	Ascomycota	Pezizomycetes	Pezizales	Pezizaceae	Peziza	P. buxea	cmp/dgst		11.1	Sap-Symb
fOTU100	Ascomycota	Sordariomycetes	Sordariales	Lasiosphaeriaceae	Apodus	A. eciduus	ctrl		68.5	Sap
fOTU45	Ascomycota	Sordariomycetes	Hypocreales	Nectriaceae			ctrl		51.9	Path-Sap-Symb
fOTU184	Ascomycota	Sordariomycetes	Hypocreales	Clavicipitaceae	Metarhizium		ctrl		42.6	Path-Sap
fOTU11	Basidiomycota	Tremellomycetes	Trichosporonales	Trichosporonaceae			ctrl	module 1	85.2	
fOTU167	Ascomycota						ctrl		63.0	
fOTU284	Blastocladiomycota						ctrl		37.0	
fOTU375	Ascomycota	Dothideomycetes	Pleosporales	Sporormiaceae			ctrl	module 1	31.5	Sap

Only taxa with significant changes in relative abundance (adjusted $p < 0.05$) are shown. 'ref', 'cmp' and "dgst" denote reference, compost or digestate fertiliser regimes, respectively. Module assignment refers to co-occurrence network modules. Prevalence denotes the proportion of the 56 surveyed farms at which the OTU was detected. Trophic modes are assigned based on FUNGuild: 'Path', 'Sap', and 'Symb' denote pathotrophs, saprotrophs, and symbiotrophs, respectively.

Table S7 Output of structural equation model including bacterial community traits.

Dependent variable	Op.	Explanatory variable	Stand. est.	Stand. err.	z-value	p-value	lower conf. int.	upper conf. int.
BR	~	Corg	0.852	0.037	23.122	0.000	0.780	0.924
BR	~	ph	0.243	0.064	3.781	0.000	0.117	0.369
BR	~	MAP	-0.245	0.071	-3.459	0.001	-0.385	-0.106
BR	~	MAT	-0.231	0.070	-3.274	0.001	-0.369	-0.093
MWD	~	ph	-0.121	0.079	-1.522	0.128	-0.277	0.035
MWD	~	BD	-0.405	0.087	-4.671	0.000	-0.575	-0.235
MWD	~	bMBD	0.227	0.080	2.843	0.004	0.070	0.383
MWD	~	clay	0.496	0.081	6.128	0.000	0.338	0.655
MWD	~	MAT	-0.165	0.078	-2.109	0.035	-0.319	-0.012
bPCoA	~	clay	-0.139	0.056	-2.500	0.012	-0.248	-0.030
bPCoA	~	cmp	-0.156	0.085	-1.837	0.066	-0.321	0.010
Corg	~	clay	0.756	0.045	16.918	0.000	0.668	0.843
Corg	~	cmp	0.198	0.060	3.280	0.001	0.080	0.316
ph	~	cmp	0.168	0.103	1.636	0.102	-0.033	0.369
BD	~	clay	-0.469	0.100	-4.676	0.000	-0.665	-0.272
bPCoA	~~	ph	-0.813	0.043	-18.836	0.000	-0.898	-0.728
ph	~~	BBlinks	-0.560	0.083	-6.733	0.000	-0.724	-0.397
bPCoA	~~	bMBD	-0.350	0.069	-5.048	0.000	-0.486	-0.214
bPCoA	~~	BBlinks	0.447	0.106	4.220	0.000	0.239	0.654
bMBD	~~	BBlinks	0.452	0.090	5.049	0.000	0.277	0.628
Corg	~~	BD	-0.651	0.078	-8.312	0.000	-0.805	-0.498

The operator "~" denotes regressions of functional proxies, physicochemical properties, bacterial community traits, or yield on pedoclimatic conditions and compost application (cmp), while "~~" represents covariances between variables. The structural equation modelling (SEM) framework is shown in Fig. 4 of the main text. Fit statistics: χ^2 = 37.69, p = 0.392, df = 36, CFI = 0.995, TLI = 0.992, RMSEA = 0.029 (RMSEA p = 0.604; 90% CI: 0.000–0.103). R^2 values: Basal respiration = 0.798; MWD = 0.662; bPCoA = 0.051; C_org = 0.662; pH = 0.028; BD = 0.220. Variable abbreviations: BD = bulk density; MAT = mean annual temperature; MAP = mean annual precipitation; Corg = soil organic carbon; bMBD = bacterial biodiversity (Shannon index); bPCoA = index for bacterial community structure; BBlinks = bacteria-bacteria association complexity.

Table S8 Output of structural equation model fungal community traits.

Dependent variable	Op.	Explanatory variable	Stand. est.	Stand. err.	z-value	p-value	lower conf. int.	upper conf. int.
BR	~	Corg	0.811	0.042	19.462	0.000	0.729	0.892
BR	~	ph	0.262	0.062	4.233	0.000	0.141	0.384
BR	~	fMBD	0.132	0.060	2.197	0.028	0.014	0.250
BR	~	MAP	-0.241	0.068	-3.566	0.000	-0.374	-0.109
BR	~	MAT	-0.207	0.068	-3.053	0.002	-0.340	-0.074
MWD	~	ph	-0.201	0.081	-2.470	0.013	-0.360	-0.041
MWD	~	BD	-0.236	0.100	-2.368	0.018	-0.432	-0.041
MWD	~	fPCoA	-0.350	0.093	-3.783	0.000	-0.531	-0.169
MWD	~	FFlinks	0.220	0.081	2.704	0.007	0.060	0.379
MWD	~	clay	0.574	0.079	7.250	0.000	0.419	0.729
MWD	~	MAT	-0.139	0.078	-1.778	0.075	-0.292	0.014
fPCoA	~	cmp	0.157	0.113	1.384	0.166	-0.065	0.379
fMBD	~	cmp	0.401	0.110	3.658	0.000	0.186	0.615
FFlinks	~	clay	-0.231	0.092	-2.519	0.012	-0.410	-0.051
FFlinks	~	cmp	0.668	0.069	9.718	0.000	0.533	0.803
Corg	~	clay	0.742	0.043	17.291	0.000	0.657	0.826
Corg	~	cmp	0.205	0.061	3.362	0.001	0.085	0.324
ph	~	cmp	0.282	0.123	2.294	0.022	0.041	0.522
BD	~	clay	-0.431	0.091	-4.714	0.000	-0.610	-0.252
fPCoA	~~	Corg	-0.539	0.094	-5.715	0.000	-0.723	-0.354
fPCoA	~~	ph	-0.216	0.105	-2.048	0.041	-0.423	-0.009
fPCoA	~~	BD	0.520	0.097	5.372	0.000	0.330	0.710
fMBD	~~	FFlinks	0.395	0.115	3.441	0.001	0.170	0.620
Corg	~~	BD	-0.653	0.078	-8.352	0.000	-0.806	-0.499
BR	~	Corg	0.811	0.042	19.462	0.000	0.729	0.892
BR	~	ph	0.262	0.062	4.233	0.000	0.141	0.384
BR	~	fMBD	0.132	0.060	2.197	0.028	0.014	0.250

The operator "~" denotes regressions of functional proxies, physicochemical properties, fungal community traits, or yield on pedoclimatic conditions and compost application (cmp), while "~~" represents covariances between variables. The structural equation modelling (SEM) framework is shown in Fig. 4 of the main text. Fit statistics: χ^2 = 35.69, p = 0.436, df = 35, CFI = 0.998, TLI = 0.996, RMSEA = 0.019 (RMSEA p = 0.642; 90% CI: 0.000–0.100). R^2 values: BR = 0.817; MWD = 0.680; fPCoA = 0.025; fMBD = 0.161; FFlinks = 0.446; C_org = 0.644; pH = 0.079; BD = 0.186. Variable abbreviations: BD = bulk density; MAT = mean annual temperature; MAP = mean annual precipitation; C_org = soil organic carbon; fMBD = fungal biodiversity (Shannon index); fPCoA = index for fungal community structure; FFlinks = fungal–fungal association complexity.

Table S9 Output of structural equation model including top modules.

Dependent variable	Op.	Explanatory variable	Stand. est.	Stand. err.	z-value	p-value	lower conf. int.	upper conf. int.
BR	~	Corg	0.775	0.051	15.075	0.000	0.675	0.876
BR	~	ph	0.222	0.064	3.436	0.001	0.095	0.348
BR	~	Module4	-0.145	0.072	-2.017	0.044	-0.285	-0.004
BR	~	MAP	-0.213	0.069	-3.102	0.002	-0.348	-0.078
BR	~	MAT	-0.178	0.070	-2.538	0.011	-0.316	-0.041
MWD	~	ph	-0.200	0.100	-1.988	0.047	-0.396	-0.003
MWD	~	BD	-0.285	0.091	-3.136	0.002	-0.463	-0.107
MWD	~	Module1	0.602	0.135	4.468	0.000	0.338	0.867
MWD	~	Module2	0.839	0.211	3.981	0.000	0.426	1.253
MWD	~	Module3	0.797	0.228	3.488	0.000	0.349	1.244
MWD	~	clay	0.382	0.093	4.120	0.000	0.200	0.563
Module1	~	MAT	-0.239	0.121	-1.981	0.048	-0.476	-0.003
Module2	~	clay	0.542	0.073	7.411	0.000	0.399	0.686
Module2	~	MAT	-0.196	0.084	-2.345	0.019	-0.360	-0.032
Module3	~	clay	-0.367	0.076	-4.846	0.000	-0.516	-0.219
Module3	~	MAT	0.235	0.087	2.693	0.007	0.064	0.406
Module4	~	clay	-0.367	0.092	-4.005	0.000	-0.547	-0.187
Module4	~	MAT	0.286	0.104	2.735	0.006	0.081	0.490
Corg	~	clay	0.758	0.044	17.355	0.000	0.672	0.843
Corg	~	cmp	0.200	0.058	3.431	0.001	0.086	0.315
ph	~	cmp	0.322	0.090	3.573	0.000	0.145	0.498
BD	~	clay	-0.469	0.100	-4.675	0.000	-0.665	-0.272
Module1	~~	Corg	0.138	0.098	1.401	0.161	-0.055	0.330
Module2	~~	Corg	0.281	0.100	2.812	0.005	0.085	0.477
Module3	~~	Corg	-0.227	0.092	-2.460	0.014	-0.408	-0.046
Module4	~~	Corg	-0.272	0.112	-2.419	0.016	-0.492	-0.052
Module1	~~	ph	0.250	0.100	2.504	0.012	0.054	0.446
Module2	~~	ph	0.593	0.082	7.238	0.000	0.432	0.753
Module3	~~	ph	-0.658	0.073	-9.055	0.000	-0.800	-0.516
Module4	~~	ph	-0.336	0.109	-3.086	0.002	-0.550	-0.123
Module2	~~	BD	-0.275	0.099	-2.766	0.006	-0.470	-0.080
Module3	~~	BD	0.151	0.087	1.724	0.085	-0.021	0.322
Module4	~~	BD	0.217	0.106	2.050	0.040	0.010	0.425
Module1	~~	Module3	-0.516	0.063	-8.159	0.000	-0.640	-0.392
Module1	~~	Module4	-0.534	0.083	-6.403	0.000	-0.697	-0.370
Module2	~~	Module3	-0.769	0.047	-16.396	0.000	-0.861	-0.677
Module2	~~	Module4	-0.508	0.085	-6.007	0.000	-0.674	-0.342
Module3	~~	Module4	0.451	0.095	4.760	0.000	0.266	0.637
Corg	~~	BD	-0.645	0.079	-8.215	0.000	-0.799	-0.491

The operator "~" denotes regressions of functional proxies, physicochemical properties, microbial module traits, or yield on pedoclimatic conditions and compost application (cmp), while "~~" represents covariances between variables. The structural equation modelling (SEM) framework is shown in Fig. 4 of the main text. Fit statistics: $\chi^2 = 31.51$, $p = 0.491$, df = 32, CFI = 1.000, TLI = 1.002, RMSEA = 0.000 (RMSEA $p = 0.683$). AIC = 895.32; BIC = 992.78. R^2 values: BR = 0.813; MWD = 0.691; Module 1 = 0.057; Module 2 = 0.311; Module 3 = 0.172; Module 4 = 0.195; C_org = 0.666; pH = 0.104; BD = 0.220. Variable abbreviations: BD = bulk density; MAT = mean annual temperature; MAP = mean annual precipitation; C_org = soil organic carbon; Module1–4 = relative abundance of microbial modules 1–4.

Supplementary References

1. Romdhane S, Spor A, Banerjee S, Breuil MC, Bru D, Chabbi A *et al.* Land-use intensification differentially affects bacterial, fungal and protist communities and decreases microbiome network complexity. *Environ Microbiome* 2022;**17**:1. https://doi.org/10.1186/s40793-021-00396-9
2. Banerjee S, Zhao C, Garland G, Edlinger A, Garcia-Palacios P, Romdhane S *et al.* Biotic homogenization, lower soil fungal diversity and fewer rare taxa in arable soils across europe. *Nat Commun* 2024;**15**:327. https://doi.org/10.1038/s41467-023-44073-6
3. Herlemann DP, Labrenz M, Jürgens K, Bertilsson S, Waniek JJ, Andersson AF. Transitions in bacterial communities along the 2000 km salinity gradient of the baltic sea. *The ISME journal* 2011;**5**:1571–79. https://doi.org/10.1038/ismej.2011.41
4. Andrews S Fastqc: A quality control tool for high throughput sequence data. Cambridge, United Kingdom.
5. Magoc T, Salzberg SL. Flash: Fast length adjustment of short reads to improve genome assemblies. *Bioinformatics* 2011;**27**:2957–63. https://doi.org/10.1093/bioinformatics/btr507
6. Schmieder R, Edwards R. Quality control and preprocessing of metagenomic datasets. *Bioinformatics* 2011;**27**:863–4. https://doi.org/10.1093/bioinformatics/btr026
7. Edgar RC. Uparse: Highly accurate otu sequences from microbial amplicon reads. *Nat Methods* 2013;**10**:996–8. https://doi.org/10.1038/nmeth.2604
8. Pruesse E, Quast C, Knittel K, Fuchs BM, Ludwig W, Peplies J *et al.* Silva: A comprehensive online resource for quality checked and aligned ribosomal rna sequence data compatible with arb. *Nucleic Acids Res* 2007;**35**:7188–96. https://doi.org/10.1093/nar/gkm864
9. Gardes M, Bruns TD. Its primers with enhanced specificity for basidiomycetes--application to the identification of mycorrhizae and rusts. *Mol Ecol* 1993;**2**:113–8. https://doi.org/10.1111/j.1365-294x.1993.tb00005.x
10. Edgar RC. Search and clustering orders of magnitude faster than blast. *Bioinformatics* 2010;**26**:2460–61.
11. Koljalg U, Larsson KH, Abarenkov K, Nilsson RH, Alexander IJ, Eberhardt U *et al.* Unite: A database providing web-based methods for the molecular identification of ectomycorrhizal fungi. *New Phytol* 2005;**166**:1063–8. https://doi.org/10.1111/j.1469-8137.2005.01376.x
12. Bengtsson-Palme J, Ryberg M, Hartmann M, Branco S, Wang Z, Godhe A *et al.* Improved software detection and extraction of its1 and its2 from ribosomal its sequences of fungi and other eukaryotes for analysis of environmental sequencing data. *Methods in Ecology and Evolution* 2013;**4**:914–19. https://doi.org/10.1111/2041-210x.12073
13. Louca S, Parfrey LW, Doebeli M, New Collective A. Decoupling function and taxonomy in the global ocean microbiome. *Science* 2016;**353**:1272–7. https://doi.org/10.1126/science.aaf4507
14. Nguyen NH, Song Z, Bates ST, Branco S, Tedersoo L, Menke J *et al.* Funguild: An open annotation tool for parsing fungal community datasets by ecological guild. *Fungal Ecology* 2016;**20**:241–48. https://doi.org/10.1016/j.funeco.2015.06.006
15. Labouyrie M, Ballabio C, Romero F, Panagos P, Jones A, Schmid MW *et al.* Patterns in soil microbial diversity across europe. *Nat Commun* 2023;**14**:3311. https://doi.org/10.1038/s41467-023-37937-4
